# Ruxolitinib-Loaded Imprinted Polymeric Drug Reservoir for the Local Management of Post-Surgical Residual Glioblastoma Cells

**DOI:** 10.3390/polym15040965

**Published:** 2023-02-15

**Authors:** Alexandra-Iulia Bărăian, Bogdan-Cezar Iacob, Olga Sorițău, Ioan Tomuță, Lucia Ruxandra Tefas, Lucian Barbu-Tudoran, Sergiu Șușman, Ede Bodoki

**Affiliations:** 1 Department of Analytical Chemistry, “Iuliu Hațieganu” University of Medicine and Pharmacy, 400349 Cluj-Napoca, Romania; 2 Institute of Oncology “Prof. Dr. Ion Chiricuță”, Laboratory of Tumor Cell Biology and Radiobiology, 400015 Cluj-Napoca, Romania; 3 Department of Pharmaceutical Technology and Biopharmacy, “Iuliu Hațieganu” University of Medicine and Pharmacy, 400012 Cluj-Napoca, Romania; 4 Electron Microscopy Center, Babes-Bolay University, 400006 Cluj-Napoca, Romania; 5 Department of Morphological Sciences, “Iuliu Hațieganu” University of Medicine and Pharmacy, 400012 Cluj-Napoca, Romania; 6 Department of Pathology, IMOGEN Research Centre, 400349 Cluj-Napoca, Romania

**Keywords:** ruxolitinib, glioblastoma, drug reservoir, molecularly imprinted polymers

## Abstract

(1) Background: The current limitations of glioblastoma (GBM) chemotherapy were addressed by developing a molecularly imprinted polymer (MIP)-based drug reservoir designed for the localized and sustained release of ruxolitinib (RUX) within the tumor post-resection cavity, targeting residual infiltrative cancerous cells, with minimum toxic effects toward normal tissue. (2) Methods: MIP reservoirs were synthesized by precipitation polymerization using acrylamide, trifluoromethacrylic acid, methacrylic acid, and styrene as monomers. Drug release profiles were evaluated by real-time and accelerated release studies in phosphate-buffered solution as a release medium. The cytotoxicity of polymers and free monomers was evaluated in vitro on GBM C6 cells using the Alamar Blue assay, optical microscopy, and CCK8 cell viability assay. (3) Results: Among the four synthesized MIPs, trifluoromethacrylic acid-based polymer (MIP 2) was superior in terms of loading capacity (69.9 μg RUX/mg MIP), drug release, and efficacy on GBM cells. Accelerated drug release studies showed that, after 96 h, MIP 2 released 42% of the loaded drug at pH = 7.4, with its kinetics fitted to the Korsmeyer–Peppas model. The cell viability assay proved that all studied imprinted polymers provided high efficacy on GBM cells. (4) Conclusions: Four different drug-loaded MIPs were developed and characterized within this study, with the purpose of obtaining a drug delivery system (DDS) embedded in a fibrin-based hydrogel for the local, post-surgical administration of RUX in GBM in animal models. MIP 2 emerged as superior to the others, making it more suitable and promising for further in vivo testing.

## 1. Introduction

Glioblastoma (GBM) is among the most complex and aggressive central nervous system tumors [1]. The defining feature of GBM is the infiltrative nature of tumor cells in the cerebral parenchyma [2]. Conventional therapy consists of the tumor’s surgical resection followed by temozolomide chemotherapy and radiotherapy [3]. A complete surgical resection of the tumor is virtually impossible because of its diffuse nature; hence, any aggressive approach can only extend survival to 12–15 months. Although therapeutic options have evolved significantly, GBM is still considered incurable to this day [4].

Signal transducer and activator of transcription (STAT-3) is a transcription factor that plays a crucial role in cell growth and survival in GBM [5,6,7,8,9]. Its activation is linked to resistance to conventional treatments and a decrease in survival of patients with GBM [10,11,12,13]. Several new molecules have been recently developed to block this signaling pathway. The FDA has approved ruxolitinib (RUX), a Janus kinase (JAK)/STAT-3 inhibitor, for the treatment of primary myelofibrosis, polycythemia vera [14], and vitiligo [15], providing clinical validation of its effectiveness in inhibiting STAT-3.

RUX has been shown to exhibit cytostatic activity in various types of cancer, including ovarian, pancreatic, colorectal, and breast cancer, as well as GBM. Studies have proven its efficacy on GBM cell cultures, making it a potential candidate for GBM chemotherapy [16]. Its ability to inactivate STAT-3 and inhibit cell proliferation, angiogenesis, and recurrence while decreasing resistance to standard therapy makes it a relevant option for GBM treatment [17,18]. However, RUX cannot cross the blood–brain barrier (BBB); thus, its systemic administration may not effectively reach brain concentrations without causing severe adverse reactions [19].

To overcome the additional obstacle represented by the BBB, several novel strategies based on local drug delivery in GBM via direct injection of anticancer drugs into the tumor resection cavity, brain parenchyma, or ventricle were developed. The most recent approach is based on the post-craniotomy administration of drug-loaded gels, nanoparticles, or polymer-based delivery systems. These drug delivery systems (DDSs) can be implanted or injected into the resection cavity and are capable of sustained drug release into the surrounding brain tissue by erosion (biodegradable) or diffusion (nonbiodegradable) mechanisms [20].

Gliadel^®^ wafer is the only commercially available topical DDS for GBM, approved by the FDA for high-grade gliomas in 1996 [21]. The implantable wafer contains 7.7 mg of carmustin, an alkylating agent, incorporated in a biodegradable polifeprosan 20 matrix [22]. Although clinical studies have shown good efficacy in both newly diagnosed and recurrent gliomas, Gliadel^®^ wafer has not become the standard of care due to high complication rates, cost, and limited availability. Furthermore, it proved to be inferior to standard temozolomide therapy in terms of survival [21].

In the last decade, various advanced drug delivery platforms have been developed using inorganic [23], organic [24], or hybrid materials [25]. Polymeric materials have emerged the most promising due to their versatility, ability to incorporate various chemotherapeutic compounds, high stability, and possibility to be administered via varied routes [26].

The molecular imprinting technique is based on the interaction of a certain template molecule of interest with one or more functional monomers and crosslinkers in order to generate a highly crosslinked 3D polymeric network. The polymerization around the template generates specific cavities that are complementary to the template in terms of shape, size, and functionality [27]. Molecularly imprinted polymers (MIPs), also known as artificial antibodies, are frequently used as recognition elements in the development of analytical methods. However, their ability to provide an extended release of a desired drug has widened their range of application to the biomedical field as drug delivery systems [28]. MIPs are of high interest, especially in oncology, since they provide improved drug release kinetics and can protect an active pharmaceutical ingredient from degradation, thus increasing its bioavailability [28,29].

MIPs are widely known for their unique features, such as high structural robustness [30], low-cost synthesis, selective binding, increased loading capacity, low immunogenicity, and possibility to be administered via various routes [27]. MIPs are considered to be highly biocompatible, especially if hydrophilic compounds are used for synthesis [31].

By designing MIPs as either drug reservoirs or targeted DDSs [32], one can successfully overcome several limitations of conventional therapies, such as the poor bioavailability of a drug or its toxic systemic effects. Additionally, MIPs offer greater flexibility for personalized chemotherapy according to an individual patient’s tumor genotype or proteomic profile, compared to the limited options offered by Gliadel^®^ wafers. MIPs can be loaded with one or several specifically selected therapeutic agents, with the additional freedom of adjusting their release kinetics [33].

**Figure 1:**
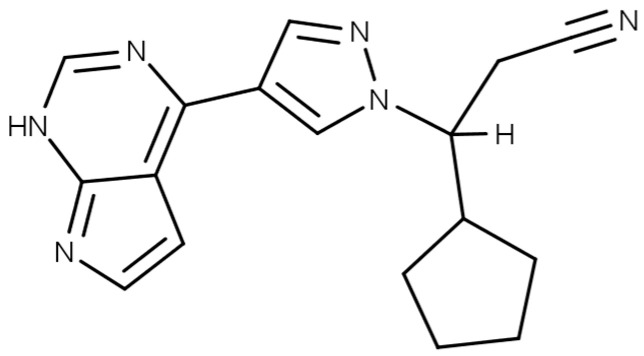
The chemical structure of ruxolitinib.

RUX, as its free base form (Figure 1), is a hydrophobic molecule (logP 2.9) with low solubility in aqueous medium (~0.1 mg/mL at 25 °C in water) [34,35]. To improve its bioavailability upon oral administration, RUX is commonly used as a phosphate salt in clinical practice, which classifies it as a BCS Class 1 drug (Biopharmaceutics Classification System) with no solubility or permeability limitations [36]. However, for the process of molecular imprinting, the free base form is preferred as it has improved solubility in organic solvents and promotes noncovalent interactions in the matrix. By loading significant amounts of RUX free base into the MIPs and administering them locally, the BBB can be bypassed, resulting in therapeutically efficient drug concentrations at the tumor site for longer periods of time.

**Figure 2:**
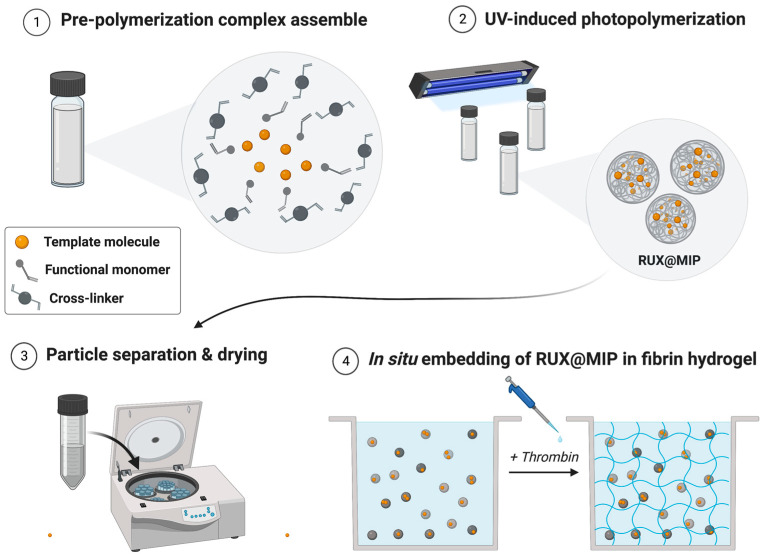
Schematic illustration of the fabrication process for the MIP-based DDS embedded in fibrin hydrogel. MIP synthesis starts with the assembly of a pre-polymerization complex using RUX, functional monomer, and crosslinker. After the UV-induced photopolymerization (24 h), RUX@MIP particles are separated by centrifugation and dried. MIP powder is the suspended in a fibrinogen solution, and the final DDS is obtained after adding thrombin to form a fibrin network; RUX@MIP = RUX-loaded MIPs. Created with BioRender.com (accessed on 19 January 2023).

The aim of the present study was to overcome the current limitations of GBM chemotherapy by designing a local MIP-based DDS for the sustained release of RUX for several days or weeks, providing high biocompatibility and potentially improved clinical outcome in the management of post-surgical residual glioblastoma cells. As such, four different MIPs were developed and thoroughly characterized by in vitro studies. A synthetic fibrin hydrogel was selected as the ideal formulation vehicle, as it is already being used to promote healing of adjacent brain tissue after tumor resection. A schematic representation of the fabrication process for the MIP-based DDS embedded into the fibrin hydrogel is presented in Figure 2.

Following the proof-of-concept demonstration on GBM cell lines, the optimized polymeric drug reservoir was planned to be administered within the tumor post-resection cavity using animal (rodent) models. The RUX-loaded polymeric DDS is intended to target the infiltrative cancerous cells responsible for the tumor recurrence by maintaining a constant therapeutical drug concentration.

## 2. Materials and Methods

### 2.1. Chemicals

Acrylamide (AM), methacrylic acid (MAA), trimethylolpropane trimethacrylate (TRIM), 2,2′-azobis(isobutyronitryle) (AIBN), temozolomide, and fibrinogen from human plasma were purchased from Sigma-Aldrich (Steinheim, Germany). Ruxolitinib (RUX) was obtained from MedChemExpress (Monmouth Junction, NJ, USA), along with trifluormethacrylic acid (TFMAA) from Alfa Aesar (Thermo Fischer, Kandel, Germany), styrene (STY) from SAFC (Sigma, Steinheim, Germany), sodium dodecylsulphate (SDS) from Redox Biovet (Cluj-Napoca, Romania), and human alpha-thrombin from Molecular Innovations (Novi, MI, USA). The following solvents were used: ethanol (EtOH), acetic acid 96% and ortho-phosphoric acid 85% from Merck (Darmstadt, Germany), acetonitrile (ACN) from Honeywell (Seltze, Germany), and phosphate-buffered solution (PBS 10×, 0.1 M, pH = 7.4) from Gibco (Thermo Fisher Scientific, Karlsruhe, Germany). Ultrapure water (18.2 MΩ, Adrona B30, Latvia) was used throughout all experiments.

For cell culture studies, F-12K medium (Kaighn’s Modification of Ham’s F-12 Medium), fetal bovine serum (FBS) (heat-inactivated, non-USA origin, sterile, filtered), horse serum (heat-inactivated, sterile, filtered), penicillin/streptomycin solution (10,000 units of penicillin, 10 mg of streptomycin/mL, sterile, filtered), L-glutamine solution (200 mM sterile, filtered), Cell Counting Kit-8 (CCK-8), and thrombin from human plasma (lyophilized powder, ≥2000 NIH units/mg protein) were purchased from Sigma-Aldrich (Taufkirchen, Germany). Alamar Blue™ Cell Viability Reagent was purchased Thermo Fisher Scientific (Karlsruhe, Germany).

### 2.2. Cytotoxicity of Free Monomers

Considering that cytotoxicity associated with polymer exposure may also be due to residual, unreacted monomers, we assessed their effect on cell viability. Chemicals tested included acrylamide (AM), trifluoromethacrylic acid (TFMAA), styrene (STY), and methacrylic acid (MAA). The minimum effective doses of RUX were assessed on the basis of increasing concentrations (75, 100, 150, and 200 µM) and further compared to a reference dose of temozolomide (50 µM).

The C6 (ATCC^®^ CCL-107™) cell line was purchased from American Type Culture Collection (ATCC) acquired through LGC Standards GmbH (Wesel, Germany). C6 is a glial tumor cell line induced by administration of N-nitrosomethylurea in rats (*Rattus norvegicus*). The clone was obtained after successive cultures and passages in animals. The culture medium consisted of F-12K Medium (Kaighn’s modification of Ham’s F-12 medium) supplemented with 2.5% FBS, 12.5% horse medium, 2 mM L-glutamine, and 1% antibiotic (penicillin + streptomycin).

To investigate the cellular response to the residual monomers, we used a cell viability test named Cell Counting Kit-8 (CCK-8), which is based on the bioreduction of WST-8 (2-(2-methoxy-4-nitrophenyl)-3-(4-nitrophenyl)-5-(2,4-disulfophenyl)-2H-tetrazolium monosodium salt) by cellular dehydrogenases to an orange formazan product that is soluble in tissue culture medium. Briefly, C6 cells were seeded in NuncTM 96-well cell culture-treated plates (Thermo Fisher Scientific, Karlsruhe, Germany) at a concentration of 10^4^ cell/well in 100 µL complete medium. Cells were allowed to adhere to culture plates. After 24 h, monomer solutions were added, obtaining different final concentrations as follows: 0.5, 0.25, 0.125, 0.062, and 0.037 mM for AM, MAA, and TFMAA; 0.5, 0.25, 0.125, and 0.062 mM for STY. RUX and temozolomide doses were also tested. After 24 h of monomer and drug exposure, 10 µL of CCK8 solution was added to each well; after 4 h of incubation, optical density was measured at 450 nm with a microplate reader Biotek Synergy 2 (Winooski, VT, USA). Statistical analysis was performed using one-way ANOVA followed by Dunnett’s multiple comparison test, using the untreated controls as references.

### 2.3. Quantitation of Ruxolitinib

Initially, the detection and continuous, real-time quantification of released RUX from the studied polymers were performed by spectrofluorimetry for further use in drug diffusion tests. More information on this topic can be found in Supplementary Material S1. Eventually, to overcome uncontrolled bias due to potential matrix effects, RUX quantitation was carried out by high-performance liquid chromatography (HPLC) with UV detection, using an Agilent 1200 chromatographic system equipped with a DAD detector. The mobile phase consisted of a 30:70 (*v*/*v*) mixture of acetonitrile (ACN) and ammonium acetate buffer (5 mM, pH = 4.5), while the stationary phase was an Agilent Eclipse XDB-C18 chromatographic column (4.6 × 150 mm, 5 μm ID). Other parameters related to the chromatographic method include 20 μL injected sample volume, 40 °C column temperature, and 0.8 mL/min flow rate, with a detection at 225 nm. For calibration, standard RUX in ethanol at different concentrations was used. Linear regression was obtained on six different levels of RUX in the range of 0.1–75 μg/mL (0.3–245 μM), injected in triplicate. All samples were filtered prior to injection using 4 mm syringe filters with 0.2 µm PTFE membranes (Phenomenex, CA, USA).

### 2.4. Synthesis of Fibrin Hydrogel

Fibrin hydrogel was selected as a vehicle for the MIP-based formulation, due to its wide clinical use as a healing aid after craniotomy. The fibrin hydrogel synthesis was conducted in situ by adding an aqueous thrombin solution (human alpha-thrombin 0.1 IU/µL in PBS 0.01 M) to a fibrinogen solution (20–40 mg/mL in PBS) at 4 °C, and then maintained at 37 °C for 30 min to form the fibrin network. In order to obtain a hydrogel with a suitable consistency, two different concentrations of fibrinogen were tested and compared for their in vitro drug release (Table 1). A solution-based formulation of RUX in PBS was also prepared and tested in order to evaluate the influence of the fibrin on drug release kinetics.

### 2.5. MIP Synthesis

Four different functional monomers were selected for the MIP synthesis, as a function of the template’s structural characteristics: AM for MIP1, TFMAA for MIP2, MAA for MIP3, and STY for MIP4 (Table 2). In all cases, TRIM was used as a crosslinker and AIBN was used as a radical initiator of the polymerization. These compounds are commonly used in molecular imprinting due to their elevated imprinting efficiency, high stability, and biocompatibility.

MIP microspheres were synthesized in organic medium by precipitation polymerization. In 8 mL flasks, a RUX solution (5 mM in ACN) and a functional monomer solution (5 mM in ACN) of AM, MAA, TFMAA, or STY were mixed to form the template–monomer pre-polymerization complex. After the crosslinker TRIM (40 mM) and initiator AIBN (4–6 mg) were added, the dissolved oxygen was removed by purging for 2 min with nitrogen. The polymerization was carried out under UV light (365 nm) for 24 h, under continuous magnetic stirring (500 rpm). After 24 h, the polymeric particles were recovered by centrifugation (7830 rpm, 30 min) using an Eppendorf^®^ 5340 centrifuge (Sigma-Aldrich, Steinheim, Germany). The MIPs were dried in a convection oven (EcoCell, BMT Medical Technology, Cejil, Czech Republic) at 40 °C for 24 h. Corresponding nonimprinted polymers (NIPs) intended to be used as references were synthesized under identical conditions, but in the absence of RUX. The molar ratio of 1:1:8 for template/monomer/cross-linker was maintained in all cases.

After synthesis, MIPs were either dispersed in the fibrin network upon the thrombin-induced polymerization of their fibrinogen suspension or placed in direct contact with the receiving medium for accelerated release experiments.

### 2.6. MIP Characterization

#### 2.6.1. Particle Size, Surface Charge, and Morphology

The particle size and zeta potential were evaluated by dynamic light scattering (DLS) using a Zetasizer Nano ZS90 (Malvern Panalytical, Malvern, UK) equipment at 25 ± 0.1 °C. Autocorrelation was measured at 90° while the laser operated at 633 nm. Size distribution was obtained from the instrumental data fitting by the inverse “Laplace transformation” and Contin methods. The polydispersity index was used as a measure of the size distribution, where values below 0.3 suggest a homogenous distribution [37]. Zeta potentials were calculated by the instrument’s software by means of the Helmholtz–Smoluchosky equation. The samples were prepared by suspending MIP/NIP powders in ethanol (1 mg/mL), followed by a 1:100 dilution in ultrapure water. All analyses were repeated in triplicate and expressed as the mean ± standard deviation.

For particle morphology assessment by scanning electron microscopy (SEM) characterization, the polymers (MIPs and NIPs) were sputter-coated with 7 nm of gold in a Polaron E–5100 plasma-magnetron sputter coater (Polaron Equipment Ltd., Watford, UK) in the presence of argon (45 s at 2 kV and 20 mA). Ultrastructural images were obtained in a Hitachi SU8320 scanning electron microscope (Hitachi, Chiyoda, Japan) at 30 kV and different magnification powers.

#### 2.6.2. Specific and Nonspecific Binding of Polymers

The drug loading capacity (LC) of MIPs was indirectly assessed, by quantifying the amount of free RUX found in the supernatant after polymerization. Following the centrifugation of the MIP samples, the supernatants were recovered and used to quantify the amount of unloaded drug. RUX quantitation was performed by HPLC-UV, following the method described in Section 2.4. Prior to injection into the chromatographic system, the supernatant samples were further diluted 1:100 with ACN and passed through syringe filter cartridges (0.2 µm PTFE membranes, Phenomenex). All analyses were repeated in triplicate and expressed as the mean ± standard deviation. Further details on data processing may be found in Supplementary Material S3.

To evaluate the nonspecific drug binding of the nonimprinted polymers, NIP suspensions (10 mg/mL) were incubated with a RUX standard solution (5 mM in ACN) and continuously stirred for 24 h on the IKA digital loopster (25 rpm). ACN was selected for drug exposure, as it was also the porogenic solvent used for MIP synthesis. Afterward, the suspensions underwent centrifugation (7830 rpm, 30 min) to remove the supernatant. The unbound RUX concentration in the recovered solutions was assessed by HPLC-UV in order to indirectly determine its nonspecific binding to NIPs. The supernatants were diluted 1:100 with ACN, filtered through 0.2 µm PTFE membrane cartridges, and injected into the chromatographic system. All analyses were repeated in triplicate and expressed as the mean ± standard deviation. The method of calculating rebinding capacity (RC) can be found in Supplementary Material S3.

### 2.7. In Vitro Drug Release Studies

#### 2.7.1. Drug Release from Fibrin Hydrogel

Real-time drug release experiments were carried out to assess RUX diffusion from the fibrin hydrogel. Experiments were conducted using a Franz cell diffusion system (PermeGear, Bechenheim, Germany), using synthetic hydrophilic polysulfone membranes (Sigma). Franz cells offer a diffusion surface of 0.6362 cm^2^ and a 10 mL volume of receiving solution. PBS 0.01 M pH = 7.4 was used as a receiving medium. The system was maintained at 37 °C throughout experiments using a thermostatic circulation bath (Julabo Corio C-B, Julabo GmbH, Seelbach, Germany), while the receiving solution was stirred continuously (500 rpm) using a magnetic stirrer. The synthetic membrane was placed between the donor and receiver compartments of the diffusion cells and was previously maintained for 30 min in PBS for equilibration. Hydrogel synthesis was performed in situ by adding all its components into the donor compartment, followed by complete gelification at 37 °C, followed by the admixture of the RUX-loaded polymer (t_0_). Then, 50 μL aliquots of the receiving solution were taken at various timepoints (3, 6, 10, 13, 20, 30, 45, 60, 90, 120, and 150 min, and 3, 4, 5, 6, 18, 20, 24, 42, and 44 h), always replenishing with fresh receiving medium. The RUX concentration in the collected samples was assessed by HPLC-UV. Results of the replicates were expressed as the normalized cumulative drug release percentage as a function of time.

#### 2.7.2. Drug Release from MIPs

The drug release profile of RUX from MIP-loaded fibrin hydrogel was initially evaluated by real-time experiments using Franz cells online coupled with continuous spectrofluorimetric detection. Details can be found in Supplementary Material S4. Nevertheless, due to repeated interruptions caused by the formation of air bubbles underneath the membrane surface during the extended experimental times and unwanted matrix effects encountered by the dissolving fibrin hydrogel components, this method was later abandoned. However, for a more time-effective evaluation of the drug release profile, we resorted to accelerated release tests where MIPs were placed in direct contact with the receiving solution. The MIPs suspension (2 mg/mL) in the release medium (PBS supplemented with 2% (m/V) sodium dodecyl sulfate to ensure sinking conditions) was placed into 2 mL tubes and continuously stirred (25 rpm) on the IKA digital loopster up to 4 days. Two sets of experiments were conducted, the first one at pH = 7.4 and the second one at pH = 5.5 (adjusted with phosphoric acid). At regular time intervals (1, 2, 3, 6, 12, 24, 48, 72, and 96 h), suspensions were separated by centrifugation (14,000 rpm, 10 min); 200 μL samples were taken from each of the supernatant and further analyzed by HPLC-UV for RUX quantification. The collected medium was replaced with fresh medium each time. Results of the replicates were expressed as cumulative drug release percentage as a function of time. The calculation method can be found in Supplementary Material S5. Mathematical modeling for the drug release kinetics was conducted using SigmaPlot 11.0 software (Systat Software Inc., San Jose, CA, USA).

#### 2.7.3. Cell Viability Assay of Polymers

The in vitro cytotoxic effects of imprinted and nonimprinted polymers were evaluated on GBM C6 cells. Hanging inserts with permeable polycarbonate membrane (0.4 µm, 12 mmØ, Merck Millipore, Ireland) containing 300 µL of fibrin loaded with various treatments were attached to 24-well plates seeded with C6 glioma adhered cells (5 × 10^4^ cells/1000 µL medium per well). The concentrations of tested MIPs were selected so that the minimum effective concentration of ~200 µM could be reached within the 24–96 h evaluation timeframe. MIPs/NIPs (20 mg/mL) were first suspended in the fibrinogen solution (10 mg/mL solution in PBS), vortexed, and then added over thrombin (0.6 IU/insert) to form the fibrin network. UV exposure for 20 min was used to sterilize the inserts. Cells were cocultured for 24, 48, and 96 h with the polymer inserts. As positive controls, three different concentrations of free RUX (200, 150, and 100 µM) in PBS, fibrin hydrogel alone, and fibrin hydrogel loaded with RUX (200 and 100 µM) were tested.

The viability of C6 glioma cell cocultures with polymers was quantitatively assessed using the Alamar Blue^®^ Cell Viability Assay. Alamar Blue is a blue dye (resazurin) used in cell viability assays to measure cell metabolism. When added to cell cultures, it is reduced to a fluorescent compound (resorufin) by cellular mitochondrial reductases, which indicates cell viability and proliferation.

After 24 h of treatment exposure, 10% Alamar Blue dye solution was added (100 µL) to each well. After the microplates were incubated in the dark for 2 h at 37 °C, 300 µL aliquots were transferred to 96-well plates for evaluation in triplicate. The fluorescence of the samples was measured using a BioTek Synergy 2 microplate reader (excitation at 540/25 nm, emission at 620/40 nm). Cell viability assessment was repeated identically at 48 h and 96 h. Differences between untreated control and polymer cocultures were analyzed using one-way ANOVA and Dunnett’s multiple comparison test. 

Cytotoxic effects were also evaluated using phase-contrast optical microscopy. After 24, 48, and 96 h, phase-contrast images were captured with a CCD camera (Axiocam MRM) adapted to a Zeiss Axio Observer D1 inverted microscope (Carl Zeiss, Jena, Germany) and were analyzed using 4.6.3 Axiovision software (Supplementary Material S6).

### 2.8. Statistical Analysis

Unless otherwise stated, statistical data analysis was performed using GraphPad Prism 8.3.1 software (San Diego, CA, USA).

## 3. Results and Discussion

### 3.1. Cytotoxicity of Free Monomers

There are several factors to consider when selecting the functional monomers for the molecular imprinting process. The monomer should have good affinity toward the targeted template, which can be achieved by choosing molecules that have complementary chemical functionalities to the drug [38].

Four different functional monomers were selected to exploit different types of noncovalent interactions within this study, namely, TFMAA and MAA as acidic monomers for their ability to form electrostatic bonds with RUX and AM for hydrogen bonds, and STY for π–π interactions. Acetonitrile was selected as a porogenic solvent, ensuring the proper solubilization of RUX free base.

Most of the studies performed on reservoir-type DDSs based on MIPs promote the idea of template elution after imprinting, followed by subsequent rebinding of the payload. Although such an approach is more favored by material scientists as it may enable a more thorough physicochemical characterization of the polymeric material; however, from an applied biomedical perspective, this would seem to be an overcomplication, i.e., a more tedious and less cost-effective process, generating hazardous wastes, especially when working with high-risk chemotherapeutic agents. Although template removal upon the imprinting step could eliminate most of the unbound drug fraction and potentially unreacted reagents, this method is also prone to leaving behind traces of eluting solvents. As an alternative, the immediate use of MIPs right after synthesis was attempted. In this case, however, the effects of the residual, unreacted monomers might need further evaluation. Considering the worst-case scenario, the remaining fraction of unreacted monomer in the dried MIPs should not be higher than a few percent following the photochemically induced radical polymerization. Therefore, the cytotoxicity of all functional monomers was investigated in the range of 0.75–10% of their molarity used for synthesis (5 mM).

**Figure 3:**
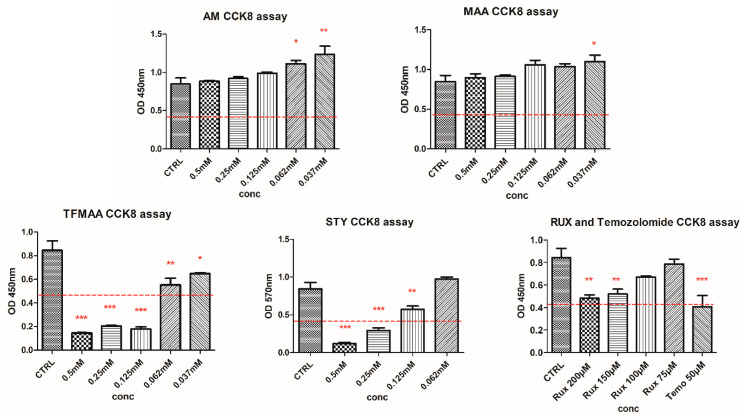
Graphical representation of CCK8 viability assay of C6 glioma cells exposed to different monomers (*** *p* < 0.001; ** *p* < 0.01; * *p* < 0.05).

The CCK8 screening on monomers indicated that the most hydrophilic monomers (AM and MAA) did not induce any cytotoxicity (Figure 3). Intermediate toxicity was observed for STY and TFMAA. Free RUX (200 μM) and temozolomide (50 μM) induced a ~50% reduction in cellular viability. As such, the 200 μM RUX was established as the minimum effective concentration and further used as a reference for MIP evaluation. Moreover, a dose-dependent effect could be observed for RUX, gradually increasing from 75 μM to 200 μM.

### 3.2. Quantitation of Ruxolitinib

Throughout the study, the identity of RUX was selectively and reliably established by HPLC-UV analysis (t_R_ = 5.1 min), whereas the levels of free RUX remaining after molecular imprinting or the released percentage of RUX from the synthesized polymers were quantitatively assessed on the basis of the chromatographic data using the linear regression established in the range of 0.1–75 μg/mL (Supplementary Material S2, Table S1, Figure S3).

### 3.3. MIP Characterization

#### 3.3.1. Particle Size, Surface Charge, and Morphology

The range of hydrodynamic diameters of the synthesized polymeric DDSs assessed by DLS analysis were found in the sub-micro- and micrometer range. The smallest values were observed for MIP2 with 484 (±19.92) nm, followed by MIP4 with 1779 (±58.82) nm, MIP1 with 1780 (±44.29) nm, and finally MIP3 with 2677 (±37.07) nm. Interestingly, in terms of particle size, the differences between the nonimprinted polymers were less significant (Table 3).

**Figure 4:**
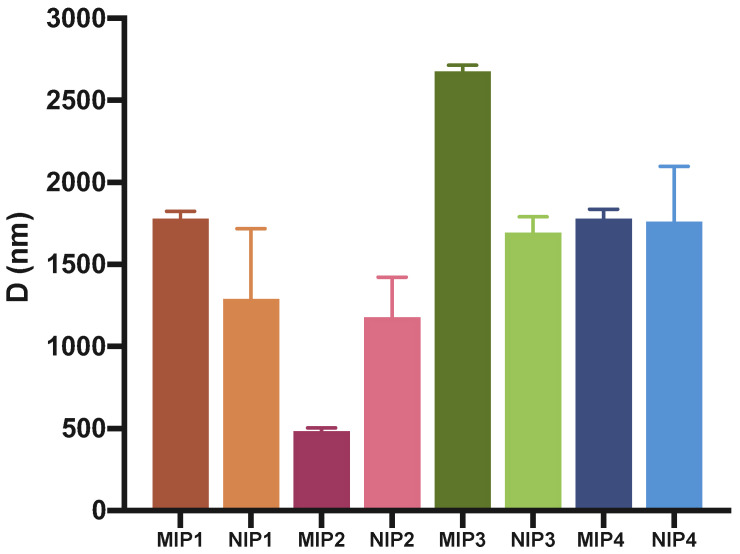
Average hydrodynamic diameter evaluated by DLS.

One of the primary characteristics that functional polymers need for potential in vivo and clinical applications is good biocompatibility, which is tightly related to their size, surface chemistry, and charge [39]. Positively charged particles seem to be more toxic as they can lead to hemolysis and clotting, compared to negatively charged or neutral polymers [39]. Taking all this into consideration, MIP2 appears to be the most promising among the synthetized polymers (all negatively charged), as it has the smallest hydrodynamic diameter and, therefore, the highest surface/volume ratio (Figure 4). The high polydispersity index (PDI) suggests the possible formation of conglomerates. This was also confirmed by SEM analysis.

**Figure 5:**
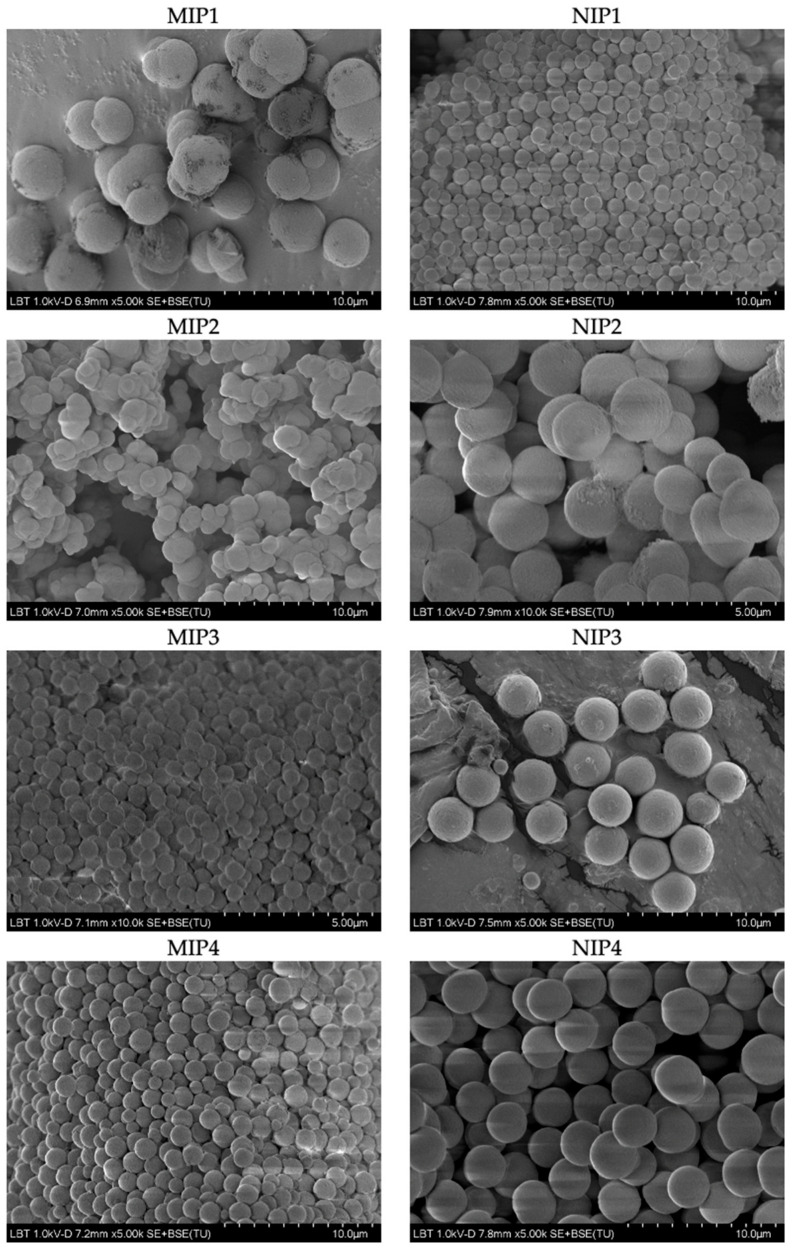
SEM images of studied MIPs and corresponding NIPs.

Particle morphology and size were evaluated also by SEM (Figure 5). The obtained micrographs confirmed the formation of conglomerates, assumed from the DLS evaluation. Whereas MIP 1, 3, and 4 were spherical and had a homogenous size distribution, MIP 2 particles had an irregular shape and a more heterogenous size distribution. Furthermore, MIP 1 had the largest particles (2–3 µm), while MIP 3 and 4 were smaller (~1 µm). In line with DLS results, MIP 2 had the smallest particles (0.4–1 µm) and, hence, the highest surface/volume ratio. This aspect is highly significant in drug delivery, since a higher ratio (surface/volume) may lead to higher drug loading and improved drug release. The nonimprinted correspondents were smaller than their imprinted counterparts, except for MIP/NIP 2. NIP 3 and 4 demonstrated the largest particles (~2 µm), followed by NIP 2 (~1.5 µm) and, finally, NIP 1 (~1 µm).

#### 3.3.2. Specific and Nonspecific Binding of Polymers

**Figure 6:**
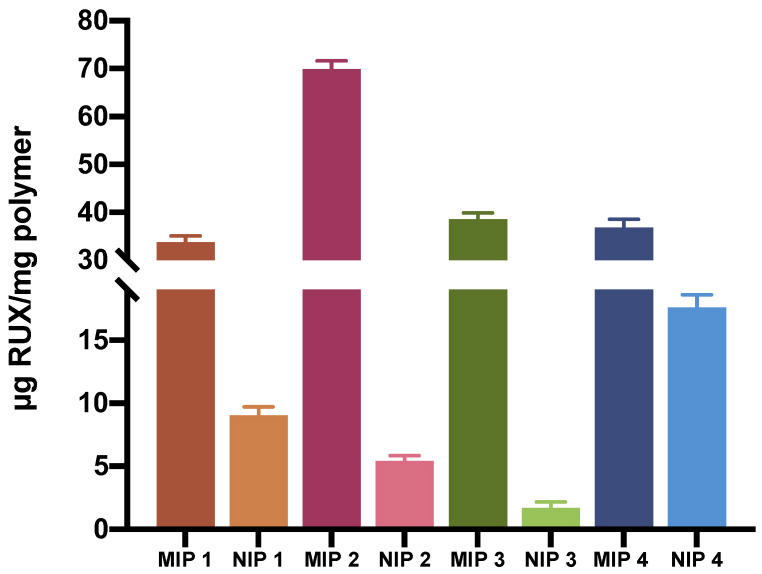
Comparison of LC of MIPs vs. RC of NIPs.

As mentioned beforehand, the MIP loading capacity (LC), involving both specific and nonspecific binding, was indirectly assessed by measuring free RUX from the supernatant solutions. MIP 2 showed the highest LC of 69.9 (±1.7), followed by MIP 3 with 38.6 (±1.3), MIP 4 with 36.9 (±1.7), and MIP 1 with 33.8 (±1.3) μg RUX/mg polymer, respectively (Figure 6).

Among all polymers, higher drug loading was observed for those based on acidic functional monomers (TFMAA and MAA), which are capable of forming ion pairs via electrostatic interactions with RUX free base. Due to its lower pKa (3.06 for TFMAA vs. 4.85 for MAA, MarvinSketch^®^ v14), TFMAA exhibits higher affinity toward RUX and, thus, a higher drug loading of the imprinted polymer.

In addition to the premises of a higher drug loading capacity, the prevalence of specific binding sites within the MIPs would be in principle responsible for the more controlled and extended drug release of the loaded drug molecule by the so-called tumbling effect [28]. Thus, binding experiments were conducted also to evaluate the level of nonspecific drug binding of the nonimprinted polymers. The following values were obtained for rebinding capacity (RC): 9.0 (±0.7) for NIP1, 5.4 (±0.4) for NIP2, 1.7 (±0.5) for NIP3, and 17.6 (±1.0) for NIP4, expressed as μg RUX/mg polymer. Higher nonspecific drug adsorption was observed for NIP 4, probably due to the more hydrophobic nature of STY. Therefore, NIP 4 could be more prone to nonspecific, hydrophobic interactions with RUX in the employed organic medium (ACN). These results further emphasize the superiority of MIPs over NIPs in terms of drug loading capacity via specific binding toward the target molecule (Figure 6), where, in the case of MIP 2, more than 92% of the total amount of loaded RUX seemed to be bound by specific interactions with the imprinted sites.

### 3.4. In Vitro Drug Release Studies

#### 3.4.1. Fibrin Hydrogel as Formulation Medium

Fibrin hydrogel is successfully used in the current clinical setting as a postoperative healing biomaterial to fill the cavity left behind by brain tumor resection, providing protection and promoting healing of nearby tissue. Fibrin hydrogel is well tolerated by the body, mimicking the structure and composition of the extracellular matrix to promote cell growth, migration, and proliferation, while simultaneously offering the ability to be used as a biocompatible, resorbable formulation matrix able to enhance treatment effectiveness [40,41,42].

**Figure 7:**
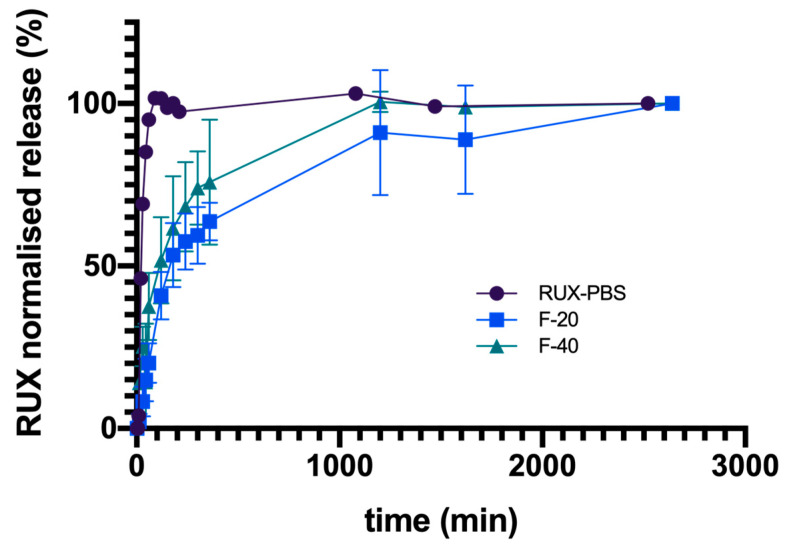
RUX normalized average cumulative transmembrane diffusion. Legend: F-20 samples contain 20 mg fibrinogen; F-40 samples contain 40 mg fibrinogen; PBS sample contains only RUX in PBS (see Table 1).

The influence of this protein matrix on the diffusion kinetics of RUX was investigated using the Franz cell assembly. The fibrin gel synthesis was conducted in situ, since its consistency could imply further difficulties in manipulation. The behavior of free RUX base embodied in fibrin-based hydrogels with different protein contents (20 mg and 40 mg fibrinogen) was monitored in comparison with RUX dissolved in PBS solution. In all cases, at the end of experiments (up to 44 h), the average cumulative percentage transfer of RUX into the receiving compartment was similar. If the transmembrane diffusion of RUX plateaued within 90 min for the RUX/PBS solution, as expected, in the case of fibrin-based hydrogels, a slower drug transfer rate into the receiving compartment was recorded, with RUX levels plateauing after ~20 h or even more (Figure 7). Interestingly, for higher concentrations of fibrinogen (40 mg) used for hydrogel synthesis, a slightly faster drug transfer was recorded. This may be related to a fibrin-facilitated membrane diffusion of the hydrophobic drug, as higher protein amounts are able to withhold and solubilize, through various H-bond and hydrophobic interactions, the dispersed RUX in the fibrin hydrogel matrix. As such, the use of a viscous fibrin-based hydrogel as the formulation vehicle for the further development and characterization of RUX-loaded MIP-based DDS in subsequent cell line studies or in vivo setups would contribute to a more desired sustained drug release profile over time.

#### 3.4.2. Drug Release from MIPs

Drug release from MIPs embedded in the fibrin hydrogel was evaluated in real time using the Franz cell system, under sink conditions ensured by the SDS-supplemented PBS release medium. As opposed to the observed drug release within the first few hours in the case of free RUX from the fibrin matrix (Figure 7), the imprinted polymers (MIP 2 and 4) embodied into the hydrogel showed a release lag time for measurable RUX of several days (Figure S6 in Supplementary Material S4). This would indicate that RUX release from the imprinted polymeric matrix into the hydrophilic compartment of fibrin hydrogel is the rate-limiting step of the DDS, having a significant contribution to the shaping of the global sustained RUX release profile. It was expected that the delay and amount of drug released from MIPs was strongly correlated with their physicochemical, structural, and morphological properties.

Throughout the real-time diffusion tests spreading over several weeks, an inconsistency in spectrofluorimetric results was observed due to the matrix effects caused by the hydrolytic erosion of the fibrin hydrogel and its slow passage into the receiving compartment. Additionally, over the long experimental study, the unavoidable formation of bubbles further contributed to the inconsistencies of the recorded drug diffusion data. More details can be found in Supplementary Material S4.

**Figure 8:**
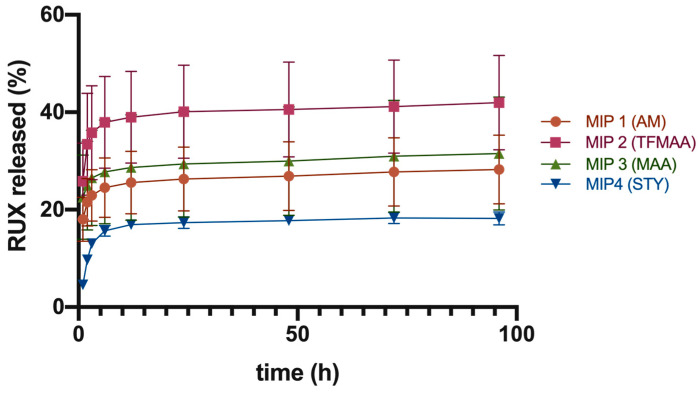
Cumulative drug release at pH = 7.4 in PBS + SDS 2%.

Given the current limitations of the abovementioned protocol, we subsequently referred to more robust analysis for comparative drug release tests from the studied imprinted polymeric scaffolds, via their direct contact with the receiving medium. The first set of experiments, conducted at a physiological pH of 7.4, showed that MIP 2 had the highest cumulative drug release of 42.0% ± 9.7% after 96 h. MIP 1, 3, and 4 showed lower cumulative drug releases of 28.2% ± 7.1%, 31.5% ± 11.6%, and 18.2% ± 1.3%, respectively (Figure 8).

Data suggested a two-phase pattern of drug release under sink conditions (Figure 8). The first phase was a quick release of the surface-adsorbed drug within the first 6–12 h, leading to a buildup of RUX levels. It is probable that RUX initially dissolved from the surface of MIPs, resulting in a “burst effect”. The second phase was a slower, sustained release phase of 18–42% of the total load over a period of 4 days at pH of 7.4.

The superior cumulative drug release from MIP 2 may be attributed its higher surface/volume ratio. The final DDS formulation could be optimized on the basis of each polymer’s loading to ensure that the minimum effective concentration of RUX (200 μm) was maintained at the administration site for an extended period of time.

**Figure 9:**
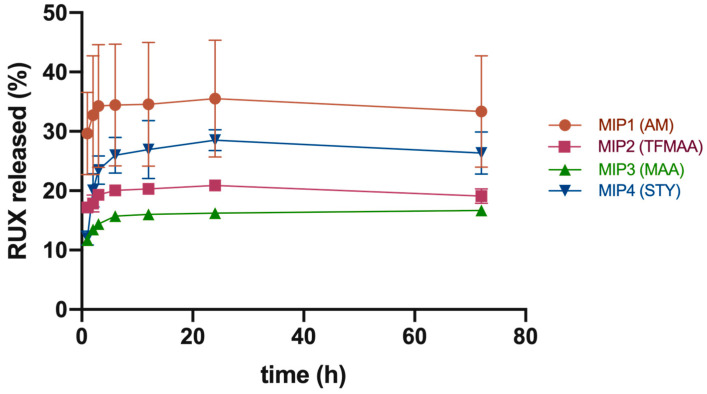
Cumulative drug release at pH = 5.5 in PBS + SDS 2%.

The second set of experiments was conducted at a pH of 5.5 (Figure 9), since it is considered that the tumor microenvironment is characterized by more acidic pH values [26]. At this pH, the cumulative percentage release values for RUX dropped by half in comparison with values obtained under physiological conditions in the case of MIPs based on acidic monomers (i.e., TFMAA and MAA) with 19.1% ± 1.2% and 16.7% ± 0.5% RUX for MIP 2 and MIP 3, respectively. For the AM-based polymer (MIP 1), values remained nearly unchanged, albeit representing the highest amount of released RUX at this pH (33.4% ± 9.4% RUX after 72 h), followed by MIP 4 (26.3% ± 3.6% RUX). The pH dependence of RUX release from the acidic acrylates was most probably related to the changes in the ionization state of these functional monomers, but steric rearrangements of the polymeric strands affecting the matrix’s permeability cannot be ruled out. Increasing the number of undissociated carboxyl moieties in the crosslinked polymeric scaffold may have led to the enhancement of noncovalent interactions (hydrophobic and hydrogen bond type bonding) with the drug molecule, impeding its release into the receiving medium. Moreover, the intermediate plateau was maintained for a shorter time, with a more evident tendency for leveling off. Nevertheless, although it is an important variable to consider in future developments of MIP-based DDSs loaded with chemotherapeutic agents, in this particular application, since the greater part of tumor is resected during surgery, the tumor microenvironment’s pH might actually be closer to the physiological value.

The drug release kinetics from the polymeric matrices was fitted with the Korsmeyer–Peppas model, in all cases obtaining a relatively good correlation (R > 0.93). Additionally, the Akaike information criterion (AIC) had the lowest values for this kinetic model, showing best fitting. The observed low values of n (n < 0.5) suggest that the drug release mechanism was mainly pseudo-Fickian diffusion (Table 4 and Table 5), which is associated with the second release phase. This is likely because the initial phase of release, referred to as the “burst” phase, was characterized by the immediate release of RUX adsorbed on the outer surface of the polymeric particles.

### 3.5. Cell Viability Assay of Polymers

**Figure 10:**
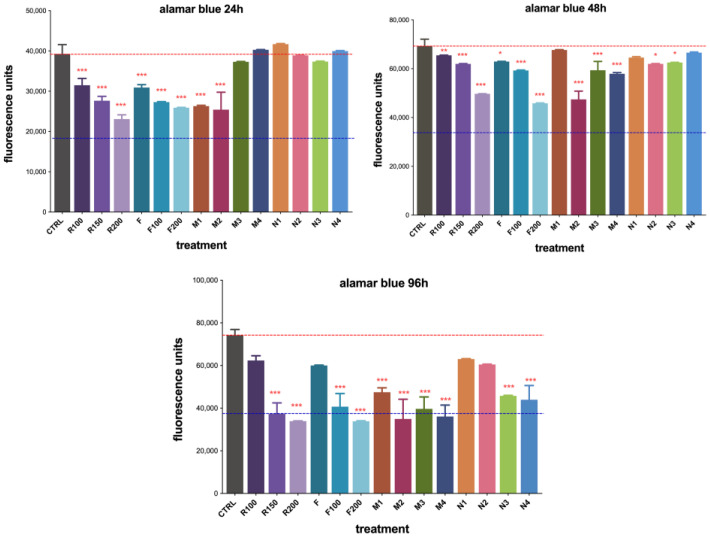
Alamar Blue viability assay of C6 glioma cells cocultured with polymers for 24, 48, and 96 h (*** *p* < 0.001; ** *p* < 0.01; * *p* < 0.05). Legend: CTRL = untreated control; R100/150/200 = free RUX in PBS 100/150/200 µM; F = plain fibrin hydrogel; F100/200 = RUX loaded fibrin gel 100/200 µM; M1–4 = RUX loaded MIPs 1–4; N1–4 = nonimprinted NIPs 1–4.

Quantitative evaluation of cell viability using the Alamar Blue assay showed a dose-dependent inhibition at all timepoints within the range of 100–200 µM by RUX in PBS (R100/150/200) or loaded into the fibrin hydrogel (F100/200), with the most pronounced effect observed at 96 h (Figure 10). MIP 1 and MIP 2 demonstrated a superior effect in reducing cell viability compared to their nonimprinted counterparts at 24 h, while no significant differences were observed for MIP/NIP 3 and MIP/NIP 4. The nonimprinted polymers showed no significant toxic effects over C6 cells (Figure 10).

After 48 h of treatment exposure, a more significant decrease in cell viability was observed for the F200 gel, confirming a given lag time in drug release offered by the protein matrix. Monomer-dependent retarded release of RUX became more evident after 48 h, with statistically significant differences in cell viability also becoming visible for MIP 3 and 4, in good correlation with data from Section 3.4.2. Additionally, the slight reduction in cell viability observed for NIPs at 48 h might have been a consequence of the nonspecific toxicity induced by the monomer residues reaching into the culture medium (Figure 10).

Cell viability significantly decreased after 96 h of treatment. As the released RUX built up, reaching the minimum therapeutic concentration, the observed cytotoxicity became statistically significant and somewhat leveled out for all studied MIPs. Surprisingly, NIPs also showed reduced cell viability, but not greater than their imprinted counterparts (Figure 10). This could be correlated with the further release of monomer residues that also influence the global cytotoxic effects of these DDSs. Although MIP 2 and 4 had similar cytotoxicity at 96 h, NIP 2 seemed to be less toxic than NIP 4 (Figure 10). Therefore, we considered MIP 2 to be the most promising for further testing. Moreover, phase-contrast optical microscopy images taken at all timepoints showed good correlation with the abovementioned quantitative analysis. Results are presented in Supplementary Material S6 (Figures S7–S9).

Free RUX offered a fast and efficient control of cell proliferation in a dose-dependent manner from the first timepoint of observation, while similar effects became obvious for the RUX-loaded imprinted polymers only after 96 h of exposure. As current cell studies could not be conducted for longer periods of time due to several limitations (i.e., high cell confluency), the beneficial effects of a sustained RUX release should be confirmed in the future using other in vitro (3D cell cultures) or in vivo (orthotropic GBM models) experimental models. Most probably, a future ideal DDS will involve a hybrid, bicompartmental hydrogel-based matrix, where free RUX should offer an initial burst release to achieve immediate therapeutical control of residual GBM cells, while the MIP-based drug reservoir should maintain therapeutic levels for a more extended period.

## 4. Conclusions

Within this study, four different MIP-based drug reservoirs intended for the local post-surgical management of residual glioblastoma cells were synthesized and characterized in vitro. Among the four types of polymeric particles obtained by precipitation polymerization in organic media, the acrylate-based polymer (MIP 2) employing TFMAA as an acidic functional monomer (MIP 2) was identified as the most suitable for further in vivo assessment for the localized release of RUX, because of its superior drug loading capacity, drug release profile, and cytotoxic efficacy on GBM cells. More than 92% of the total amount of loaded RUX seemed to be bound by specific interactions with the imprinted sites in the case of MIP 2, demonstrating once again the superior nature of molecularly imprinted polymers as drug reservoirs compared to their nonimprinted counterparts.

A two-phase release profile was observed under sink conditions. The initial release of the surface adsorbed drug occurred rapidly in the first 6–12 h, leading to the build-up of RUX levels. Subsequently, a slower-paced sustained drug release took place, culminating in the release of ~40% of the total load over the course of 4 days for MIP 2. A pH dependence of RUX release was observed in the case of imprinted polymers based on acidic functional monomers (i.e., MAA and TFMAA), which might require consideration in future applications. An additional support in extended drug release may be given by the fibrin-based hydrogel proposed as formulation medium, acting also as a second-phase pseudo-reservoir matrix. The assessment of cell viability using CCK8 assay revealed no substantial impact on cellular viability of the free monomers that may be present in trace amounts as a result of molecular imprinting. The Alamar Blue assay showed that MIP 2 exhibited superior cytotoxic efficacy on the examined GBM C6 cells.

In conclusion, an ideal DDS for the local management of residual GBM cells should consist of a hybrid material, having both free RUX loaded into the fibrin hydrogel to offer a burst release and RUX-loaded MIPs that are able to maintain a prolonged cytostatic effect. Further validation of these findings through in vivo testing using animal models is required.

## 5. Patents

The authors have intellectual property in the form of a pending patent related to the described research.

## Figures and Tables

**Table 1:** Composition of RUX-loaded fibrin hydrogel formulations.

Hydrogel Component	Amounts and Formulation Codes		
	F-20	F-40	RUX-PBS
Fibrinogen	20 mg	40 mg	-
Thrombin 0.1 IU/µL	8.75 µL	8.75 µL	-
RUX	700 µg	700 µg	700 µg
PBS pH = 7.4	300 µL	300 µL	300 µL

**Table 2:** Composition of the pre-polymerization mixtures.

Polymer	Template 5 mM	Functional Monomer 5 mM	Cross-Linker 40 mM	Initiator	Solvent
MIP 1	RUX	AM	TRIM	AIBN	ACN
MIP 2		TFMAA			
MIP 3		MAA			
MIP 4		STY			
NIP 1	-	AM			
NIP 2		TFMAA			
NIP 3		MAA			
NIP 4		STY			

ACN = acetonitrile, AM = acrylamide, AIBN = azobis(isobutyronitrile), MAA = methacrylic acid, MIP = molecular imprinted polymer, NIP = nonimprinted polymer, RUX = ruxolitinib, STY = styrene, TFMAA = trifluoromethacrylic acid, TRIM = trimethylolpropane trimethacrylate.

**Table 3:** Particle characteristics determined by DLS.

Polymer	D (nm) ± SD	PDI ± SD	ZP (mV) ± SD	Mob (μm/Vs) ± SD	Cond (mS/cm) ± SD
MIP 1	1780 ± 44.29	0.799 ± 0.074	−47.07 ± 0.404	−3.689 ± 0.033	0.03787 ± 0.00155
MIP 2	484 ± 19.92	0.584 ± 0.045	−36.57 ± 1.168	−2.867 ± 0.094	0.00453 ± 0.00433
MIP 3	2677 ± 37.07	0.873 ± 0.219	−43.00 ± 0.300	−3.369 ± 0.025	0.00582 ± 0.00212
MIP 4	1779 ± 58.82	0.637 ± 0.175	−38.70 ± 0.721	−3.035 ± 0.055	0.00473 ± 0.00284
NIP 1	1291 ± 427.76	0.920 ± 0.139	−26.50 ± 1.510	−2.073 ± 0.121	0.00849 ± 0.00894
NIP 2	1179 ± 244.25	0.929 ± 0.066	−40.60 ± 2.193	−3.172 ± 0.167	0.00950 ± 0.00631
NIP 3	1696 ± 95.35	0.831 ± 0.123	−41.5 ± 1.020	−3.250 ± 0.080	0.00880 ± 0.00970
NIP 4	1784 ± 305.31	0.935 ± 0.097	−35.10 ± 0.872	−2.751 ± 0.069	0.00305 ± 0.00212

D = hydrodynamic diameter, MIP = molecular imprinted polymer, NIP = nonimprinted polymer, PDI = polydispersity index, SD = standard deviation, ZP = zeta potential, Mob = mobility, Cond = conductivity.

**Table 4:** Drug release kinetics at pH = 7.4.

MIP	Korsmeyer-Peppas Model			
	R	AIC	k	n
1	0.9926	24.112	20.2924	0.0766
2	0.984	38.550	31.125	0.072
3	0.9971	17.465	24.031	0.0612
4	0.9307	39.123	9.6048	0.1607

**Table 5:** Drug release kinetics at pH = 5.5.

MIP	Korsmeyer-Peppas Model			
	R	AIC	k	n
1	0.9929	27.950	32.0788	0.0224
2	0.9984	4.463	17.5111	0.0607
3	0.9941	11.552	12.5756	0.0934
4	0.9664	37.564	16.7725	0.1881

## Data Availability

Not applicable.

## Supplementary Materials

The following supporting information can be downloaded at https://www.mdpi.com/article/10.3390/polym15040965/s1: Figure S1. Fluorescence spectrum of RUX; Figure S2. Spectrofluorimetry calibration curve; Figure S3. Linear regression of RUX using HPLC analysis; Figure S4. UV spectrum of RUX; Figure S5. Illustration of the semi-automated custom-made system for diffusion tests; Figure S6. Release profile of RUX from MIP 2 and MIP 4 in Franz cells; Figure S7. Phase contrast microscopy images taken at 24 h with 10X objective; Figure S8. Phase contrast microscopy images taken at 48 h with 10X objective; Figure S9. Phase contrast microscopy images taken at 96 h with 10X objective; Table S1. Chromatographic data of the calibration set of standard RUX for regression analysis; Table S2. Drug release at pH = 7.4; Table S3. Drug release at pH = 5.5.
