# Supplementary material for: Ruxolitinib-Loaded Imprinted Polymeric Drug Reservoir for the Local Management of Post-Surgical Residual Glioblastoma Cells
Source: Polymers (Basel). 2023 Feb 15;15(4):965. doi: 10.3390/polym15040965 (PMC9962605; doi:10.3390/polym15040965)
Supplement: Supplementary file 1 [file polymers-15-00965-s001.zip › polymers-2139998-supplementary.pdf]

polymers

MDPI

Supplementary Material

Ruxolitinib-loaded imprinted polymeric drug reservoir for the local management of post-surgical residual glioblastoma cells

Alexandra-Iulia Bărăian, Bogdan Cezar Iacob, Olga Sorițău, Ioan Tomuță, Lucia Ruxandra Tefas, Lucian Barbu- Tudoran, Sergiu Șușman and Ede Bodoki

S1. Spectrofluorimetric detection and quantification of RUX

Detection and quantification of RUX were initially performed by spectrofluorimetry, using an Avantes system with AvaLight-XE pulsed xenon light source and AvaSpec-ULS2048CL-EVO spectrometer. Detection was done in a low-volume quartz cuvette (100 µL). Excitation wavelength λ_{ex} = 320 nm and emission wavelength λ_{em} = 386 nm. Data collection was done using integrated AvaSoft software.

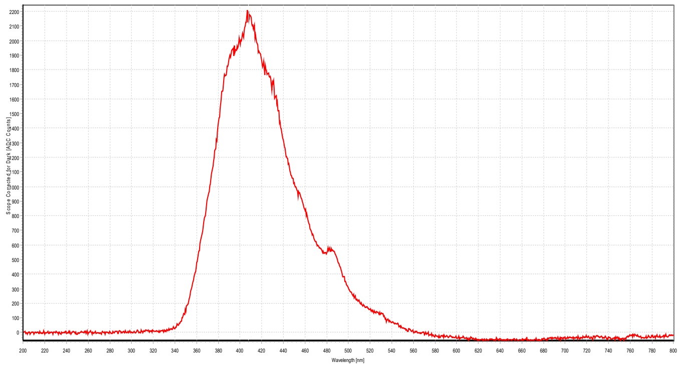

Figure S1 Fluorescence spectrum of RUX

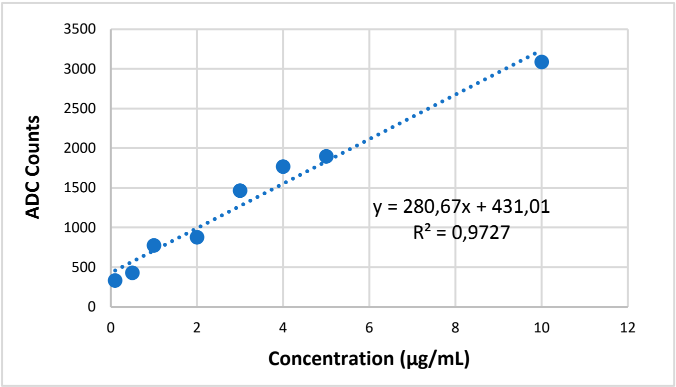

Figure S2. Spectrofluorimetry calibration curve

S2. HPLC detection and quantification of RUX

Table S1 Chromatographic data of the calibration set of standard RUX for regression analysis

C (µg/mL) RUX	Peak area			Average area	SD
	Series 1	Series 2	Series 3		
0.1	3.844	3.869	3.617	3.78	0.139
1	38.1	38.9	38.5	38.50	0.566
5	181	181.3	180.35	180.88	0.486
10	358.4	359.9	361.1	359.80	1.353
50	1594.7	1597.7	1600.9	1597.77	3.101
75	2361	2365.3	2364.4	2363.57	2.268

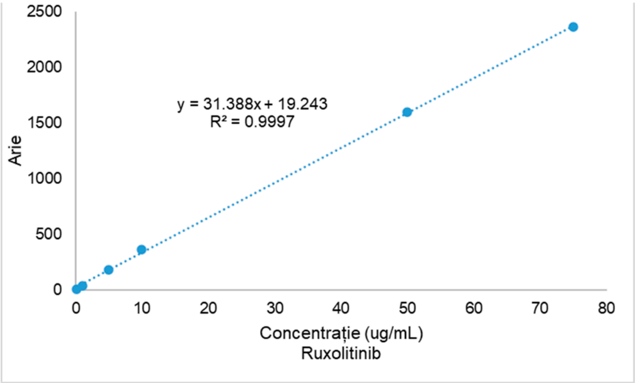

Figure S3. Linear regression of RUX using HPLC analysis

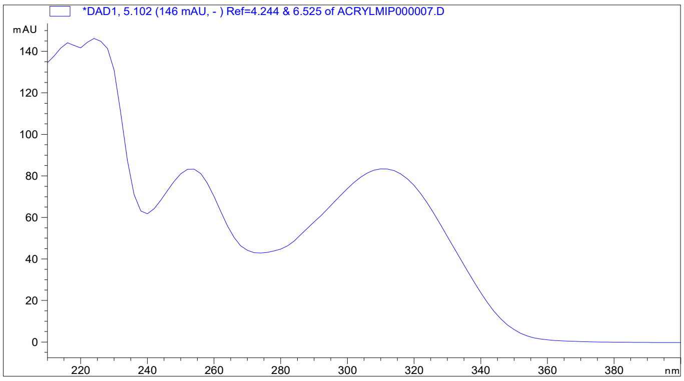

Figure S4. UV spectrum of RUX

S3. Calculations of loading capacity and rebinding capacity

RUX's concentration in the supernatant corresponds to the unloaded drug, so the quantity of MIP-loaded RUX was calculated as follows:

$$LC = \frac{m_{loaded} \times 1000}{m_{MIP}}$$

$$m_{loaded} = m_0 - m_{sup}$$

$$m_0 = V_0 \times M_M \times C_M \times 1000$$

$$m_{sup} = \frac{c_{sup} \times V_{sup}}{1000}$$

$$where$$

LC = loading capacity (mg RUX/g MIP), m_{loaded} = mass of loaded RUX (mg), m_{MIP} = mass of final MIP product (mg), m_0 = mass of total RUX from initial solution (mg), m_{sup} = mass of RUX in the supernatant solution (mg), V_0 = volume of initial solution prior to polymerization (L), M_M = molar mass of RUX (g/mole), C_M = RUX concentration in the initial solution (mole/L), c_{sup} = RUX concentration in the supernatant solution after polymerization (μg/mL), V_{sup} = volume of supernatant after polymerization (mL)

The amount of rebound RUX was calculated as follows:

$$RC = \frac{m_{rebound} \times 1000}{m_{NIP}}$$

$$m_{rebound} = m_0 - m_{sup}$$

$$m_0 = V_0 \times M_M \times C_M$$

$$m_{sup} = \frac{c_{sup} \times V_0}{1000}$$

where

RC = rebinding capacity (mg RUX/g NIP), $m_{rebound}$ = mass of rebound RUX (mg) , m_{NIP} = mass of initial amount of weighted NIP powder (mg), m_0 = total amount of RUX in the initial solution (mg), m_{sup} = amount of RUX in the supernatant solution after separation (mg), V_0 = volume (L) of initial RUX stock solution added, according to the amount of NIP for 10mg/mL, M_M = molar mass of RUX (g/mole), C_M = RUX concentration in the initial stock solution (mole/L), c_{sup} = RUX concentration in the supernatant solution after binding (μg/mL)

S4. Real-time drug diffusion tests with spectrofluorimetric detection

The drug release profile of RUX from MIPs was initially evaluated by real-time experiments using the same Franz cell-based system, coupled with spectrofluorimetric detection. The sampling process was automated by using a peristaltic pump (IsmaTec, IPC) which continuously took samples from the Franz cell and transferred them to the detection cell. Release medium consisted of PBS supplemented with 2% (m/V) sodium dodecyl sulfate. The donor compartment was loaded with 300 μL fibrin hydrogel made by suspending MIPs (5.35 mg MIP2, 3.63 mg MIP4) in a fibrinogen solution (20 mg/mL) and adding thrombin. Cumulative drug release (Figure S4.2) was assessed by spectrofluorimetry, based on the method described in S1.

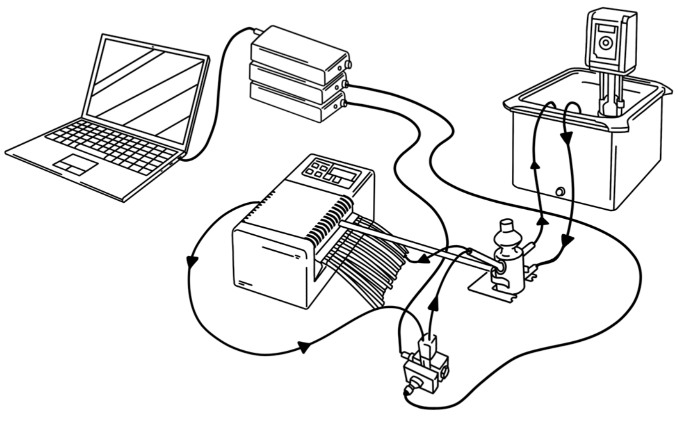

Figure S5. Illustration of the semi-automated custom-made system for diffusion tests

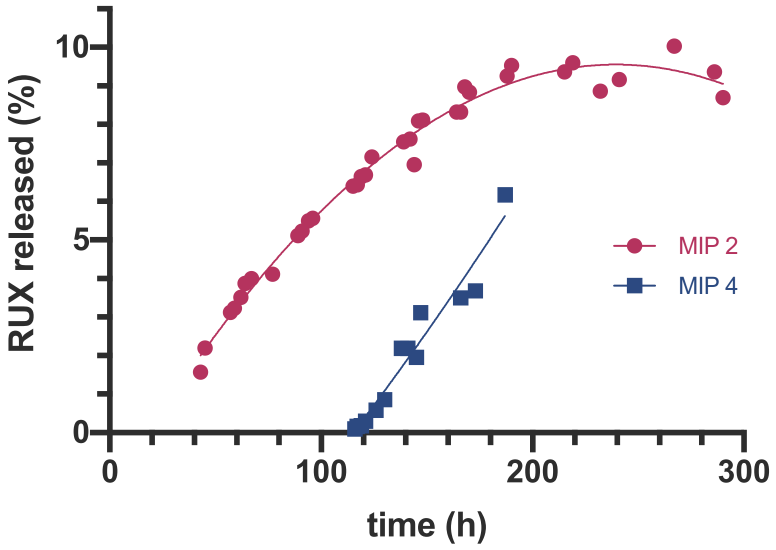

Figure S6. Release profile of RUX from MIP 2 and MIP 4 in Franz cells

S5. Accelerated drug release studies with HPLC-UV detection

Results are presented as cumulative drug release percentages, based on the following formulas:

$$\textit{For each time interval } t_i \textit{ where } i = 1 \rightarrow 9$$

$$q_i = C_i \times V_s$$

$$Q_i = C_i \times V_0 + \sum_{i=1}^{i=9} q_{i-1}$$

$$P_i = \frac{Q_i}{Q_0} \times 100$$

$$\textit{while}$$

$$V_0 = \frac{m_{MIP}}{2}$$

$$Q_0 = m_{MIP} \times LC$$

$$\textit{where}$$

t = time (hours), i = , q_i = quantity of RUX (μg) in samples at each time interval, C_i = RUX concentration in sample (μg/mL) at each time interval, V_S = volume of the sample (mL), Q_i = cumulative quantity of RUX (μg) at each time interval, V_0 = total volume of release medium (mL) as a function of MIP amount for 2mg/mL suspensions, P_i = percentage of RUX (%) released at each time interval, Q_0 = total amount of RUX found in the amount of weighted MIPs (μg), m_{MIP} = total amount of MIP weighted (mg), LC = loading capacity (μg RUX/mg MIP or mg RUX/g MIP).

Table S2. Drug release at pH = 7.4

Time (h)	RUX released (%) ± SD			
	MIP 1	MIP 2	MIP 3	MIP4
1	18.020 ± 4.480	25.860 ± 7.826	22.568 ± 8.644	4.558 ± 0.571
2	21.503 ± 4.864	33.439 ± 10.462	24.940 ± 9.091	9.691 ± 0.870
3	22.953 ± 5.295	35.785 ± 9.664	26.501 ± 9.784	13.016 ± 0.983
6	24.510 ± 6.117	37.954 ± 9.411	27.727 ± 10.657	15.662 ± 1.114
12	25.576 ± 6.428	38.999 ± 9.439	28.656 ± 10.824	16.915 ± 0.861
24	26.292 ± 6.527	40.124 ± 9.549	29.383 ± 10.906	17.357 ± 1.178
48	26.877 ± 7.036	40.575 ± 9.734	29.996 ± 11.171	17.764 ± 1.065
72	27.737 ± 7.025	41.165 ± 9.570	30.984 ± 11.458	18.284 ± 1.129
96	28.232 ± 7.072	41.998 ± 9.703	31.501 ± 11.618	18.221 ± 1.338

Table S3. Drug release at pH = 5.5

Time (h)	RUX released (%) ± SD			
	MIP 1	MIP 2	MIP 3	MIP4
1	29.662 ± 6.920	17.191 ± 0.325	11.681 ± 0.488	12.040 ± 1.172
2	32.721 ± 9.997	17.839 ± 1.445	13.476 ± 0.368	20.073 ± 2.789
3	34.272 ± 10.341	19.311 ± 0.766	14.417 ± 0.521	23.472 ± 2.392
6	34.450 ± 10.245	20.069 ± 0.119	15.746 ± 0.472	25.988 ± 3.025
12	34.574 ± 10.411	20.309 ± 0.671	16.008 ± 0.697	26.930 ± 4.896
24	35.536 ± 9.864	20.893 ± 0.903	16.227 ± 0.604	28.504 ± 1.775
72	33.364 ± 9.392	19.124 ± 1.226	16.692 ± 0.510	26.337 ± 3.569

S6. Phase contrast microscopy images

Phase contrast microscopy offers information about the number of cells, their morphology and viability. Thus, we recorded microscopy images of cell plates as part of the experiment in order to assess cell viability following exposure to RUX, MIPs and NIPs. Cytotoxicity of can be observed by a decrease in cell number and their tendency to aggregate in small colonies (Figures S6.1, S6.2, S6.3).

CTRL1

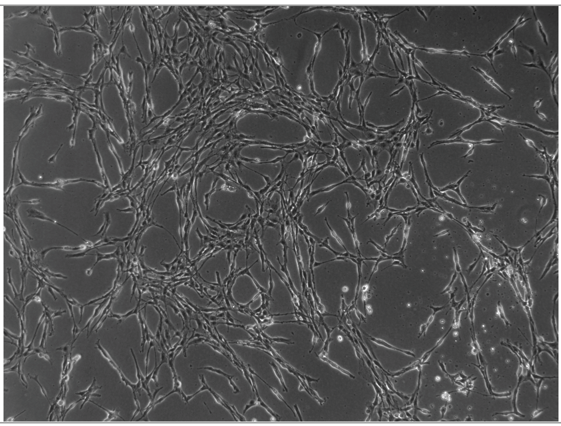

CTRL2

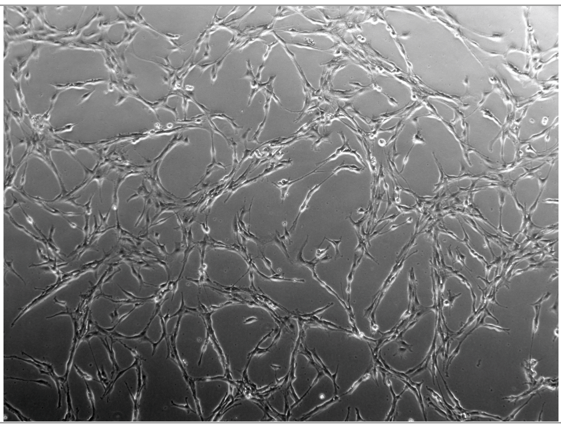

**R100**	**R150**
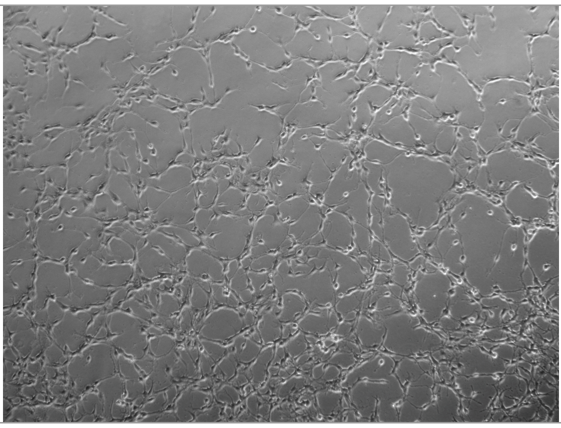	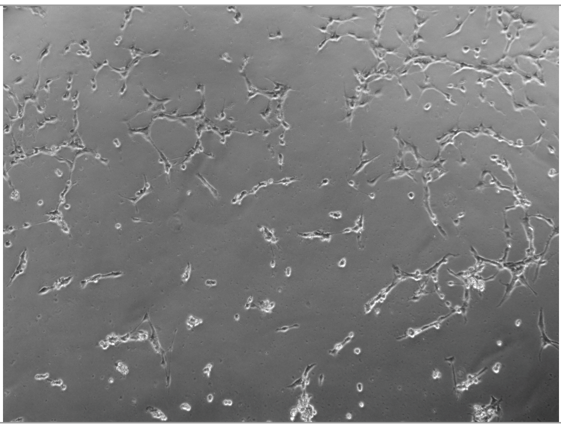
**R200**	**F**
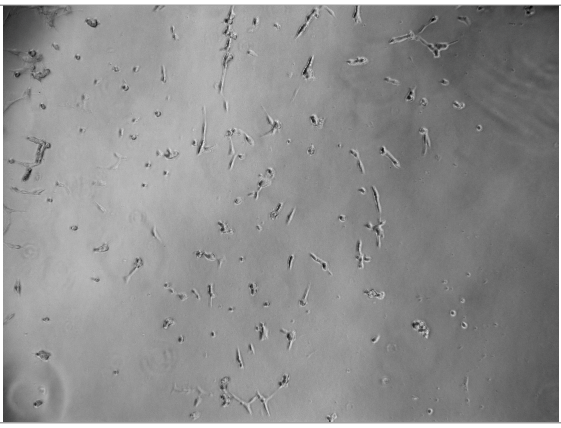	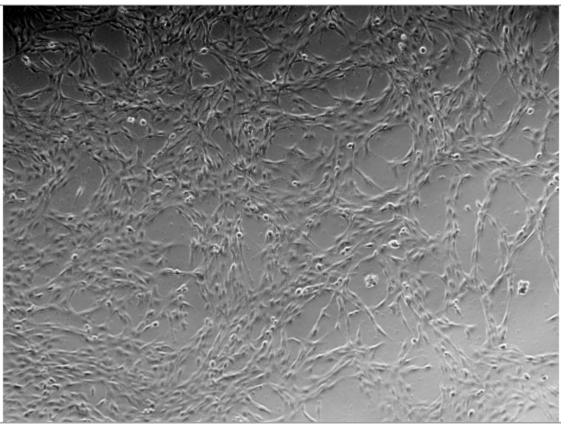
**F100**	**F200**
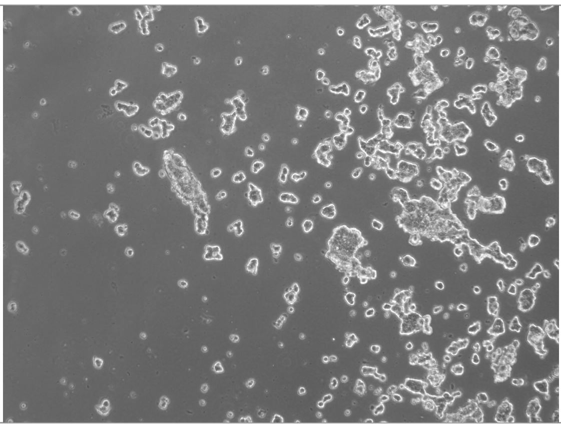	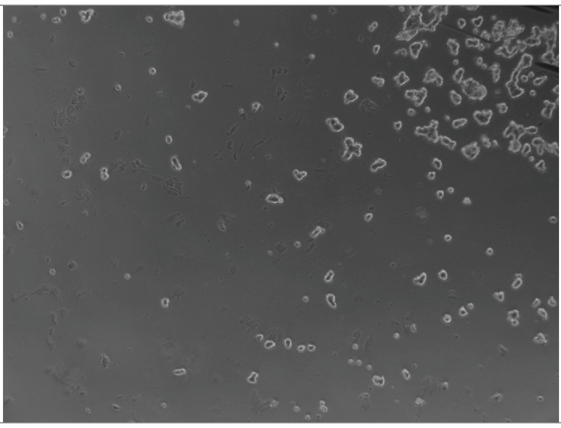
**M1**	**N1**
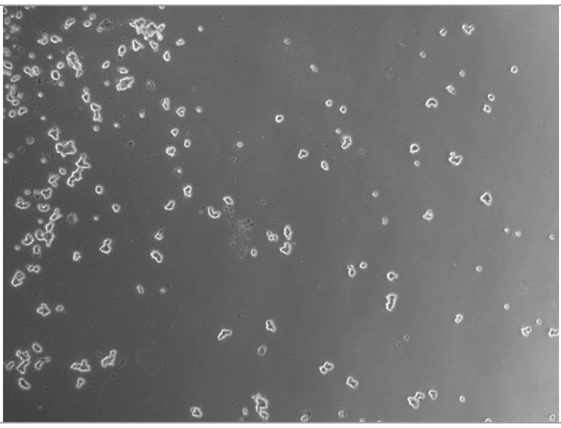	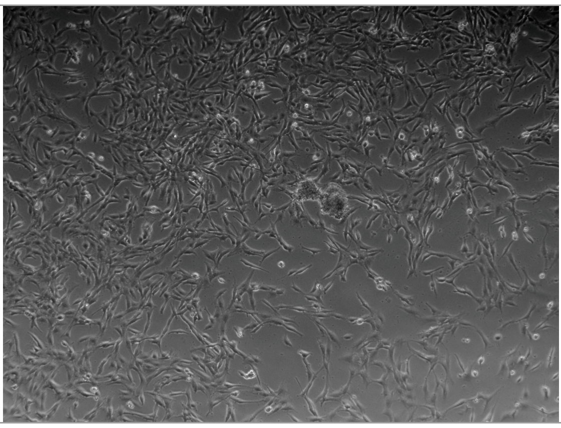

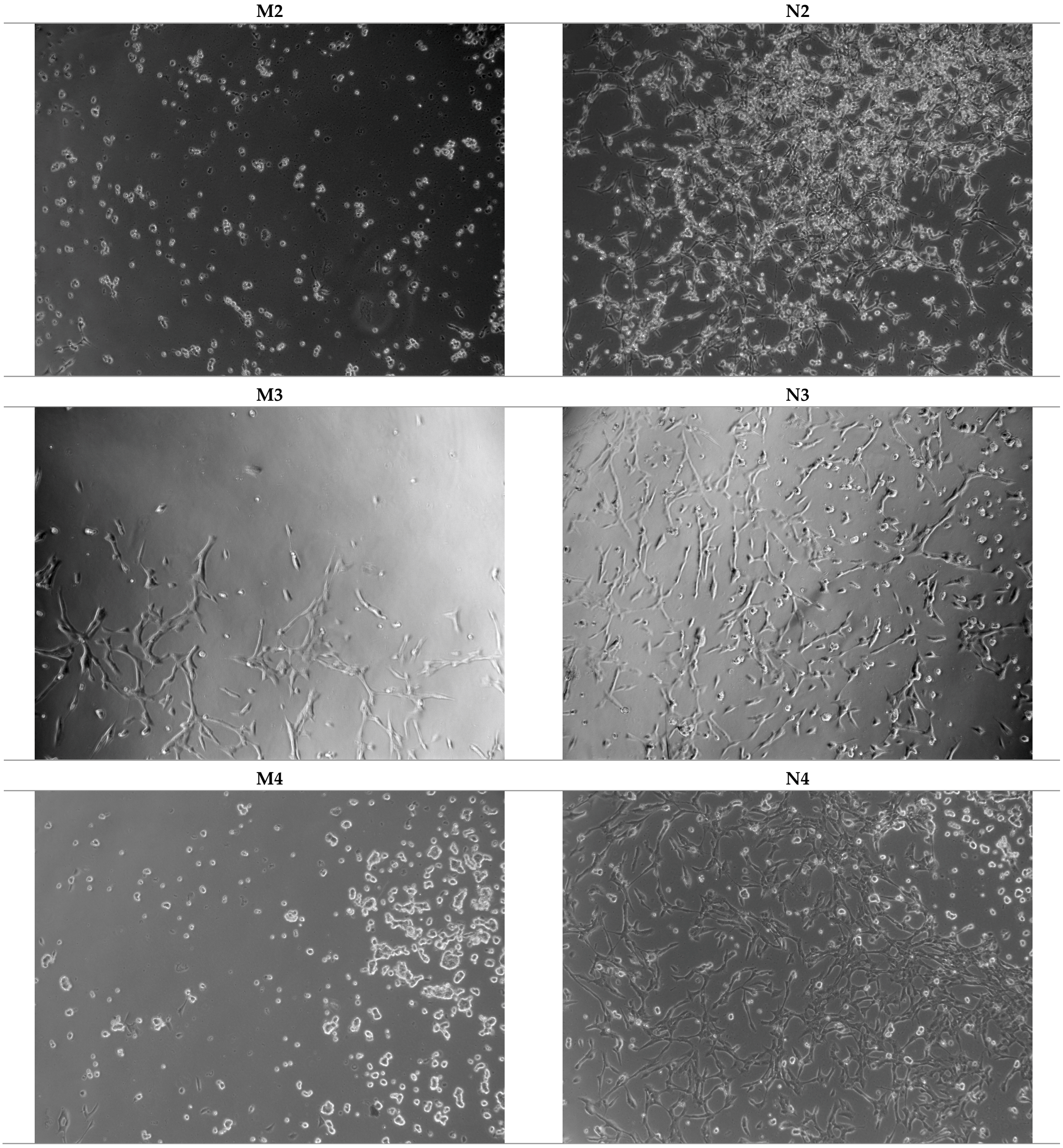

Figure S7. Phase contrast microscopy images taken at 24 hours with 10X objective

CTRL1

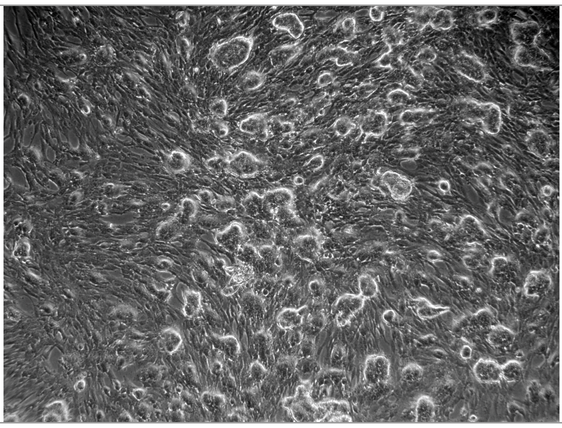

CTRL2

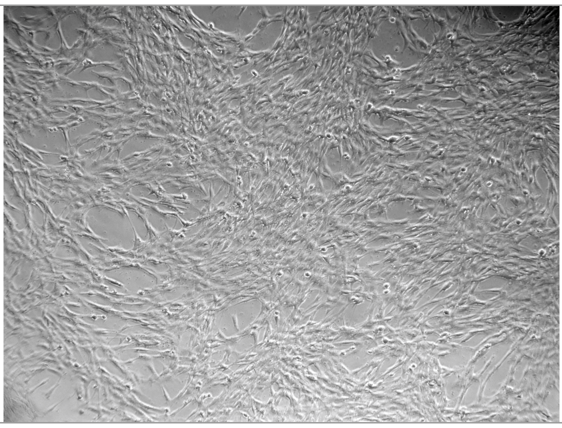

R100

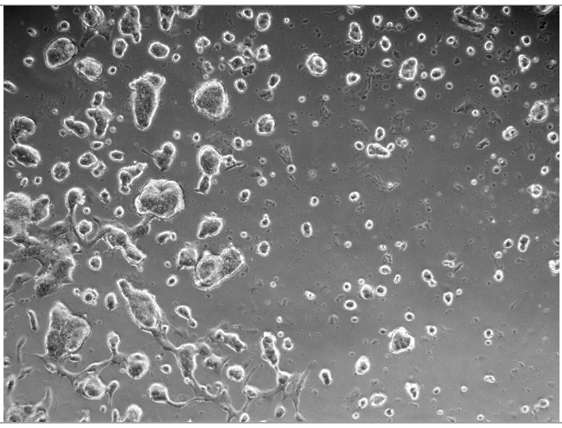

R150

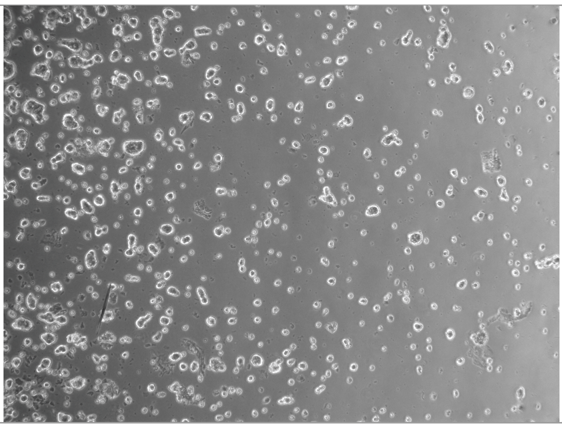

R200

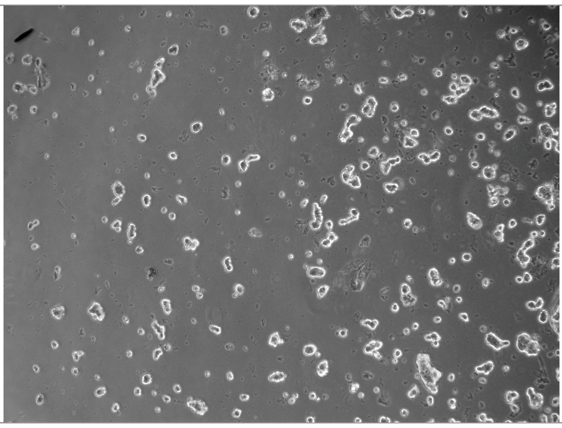

F

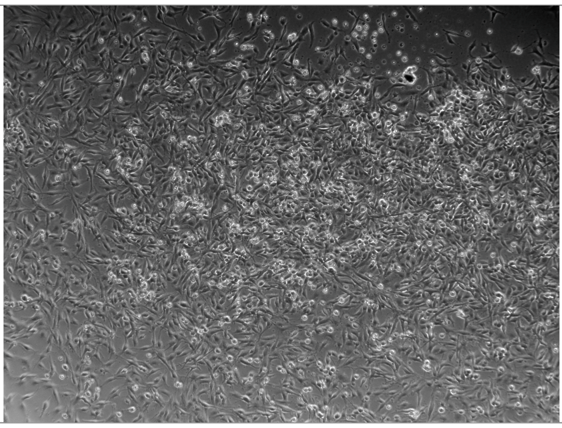

F100

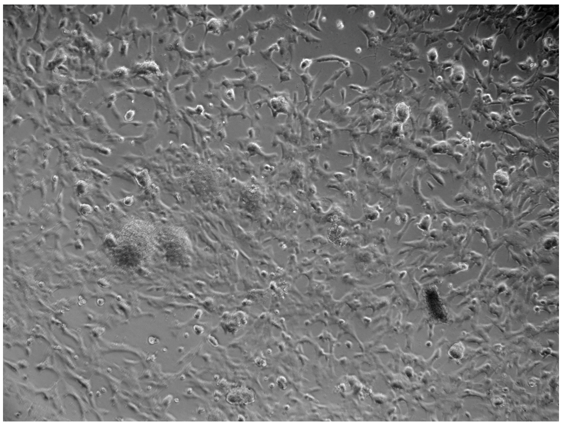

F200

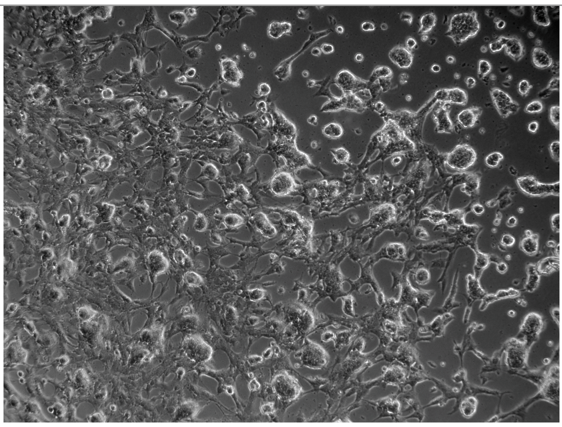

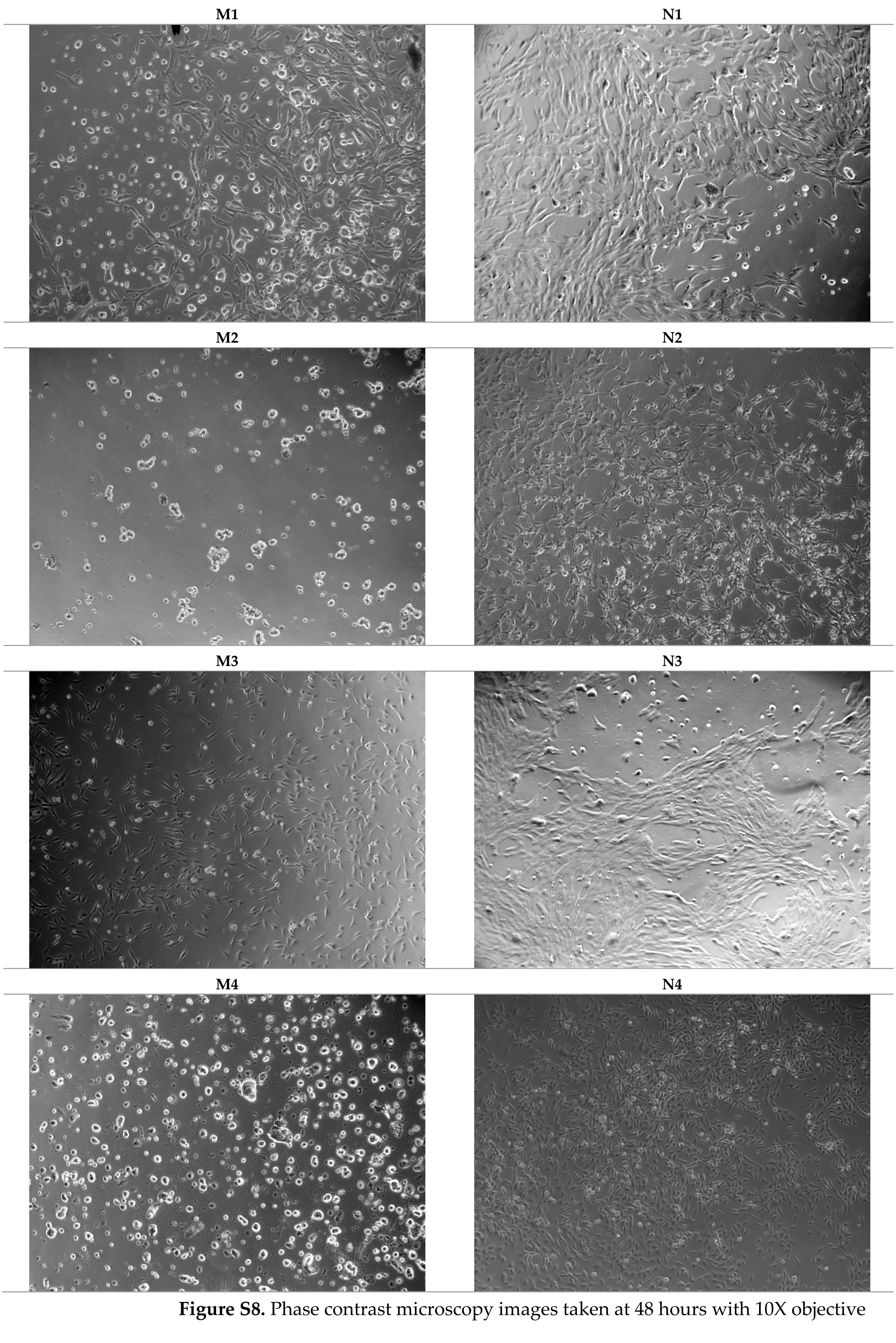

Figure S8. Phase contrast microscopy images taken at 48 hours with 10X objective

**CTRL1**	**CTRL2**
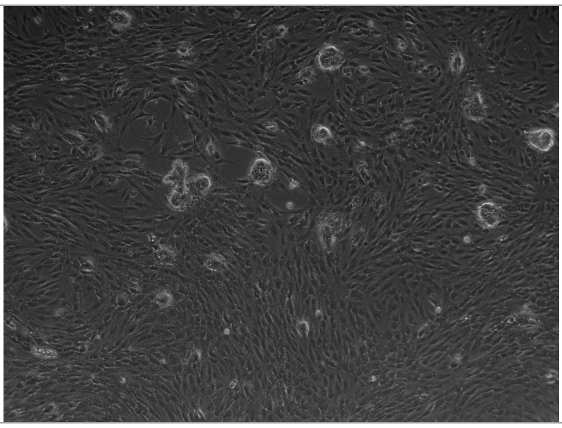	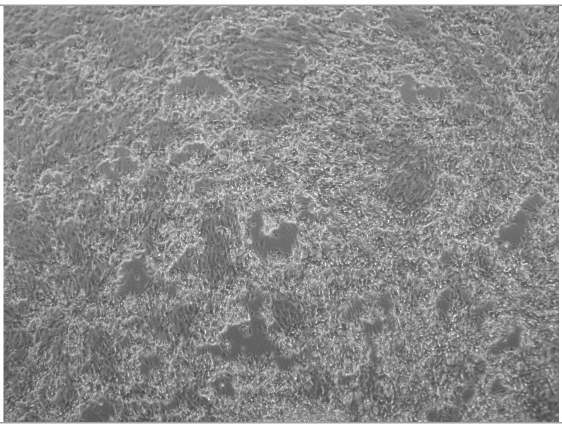
**R100**	**R150**
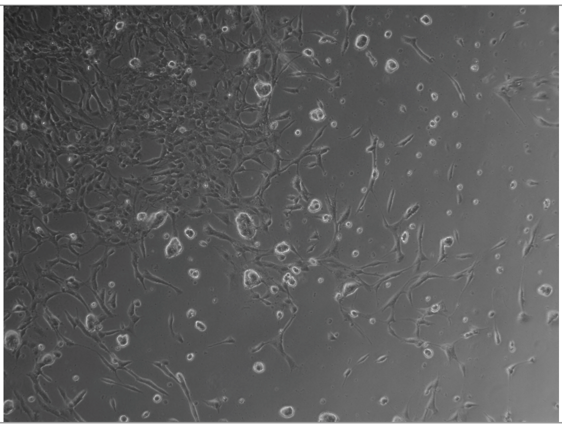	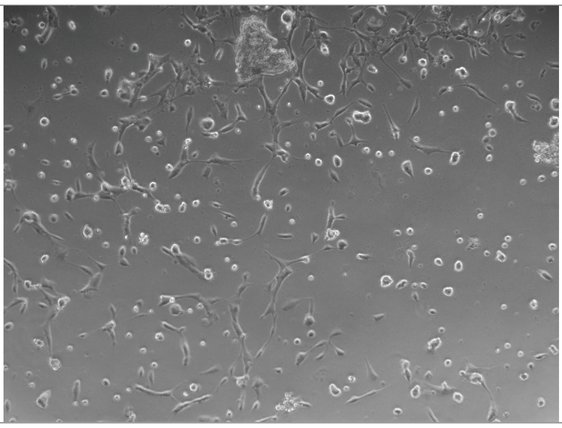
**R200**	**F**
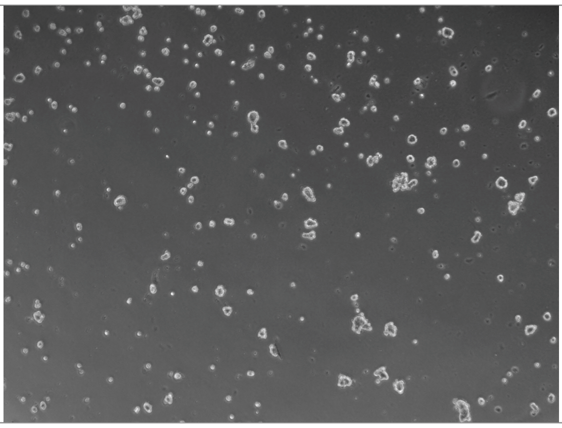	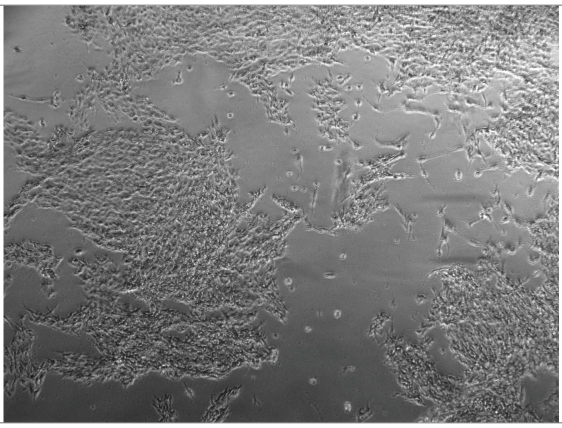
**F100**	**F200**
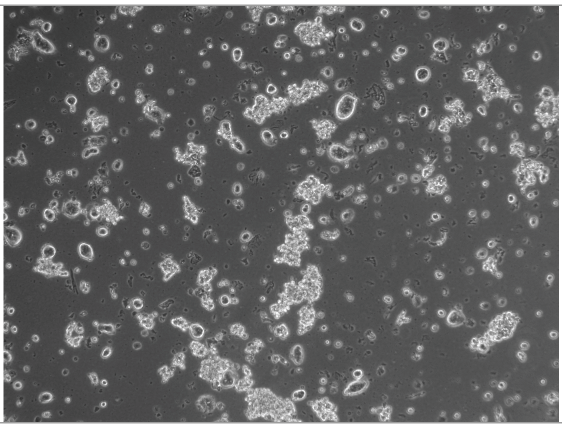	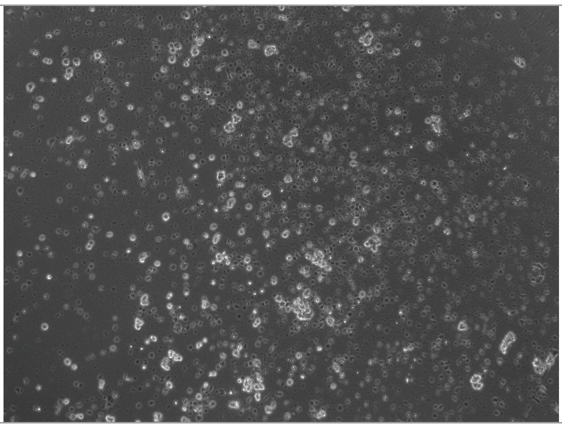

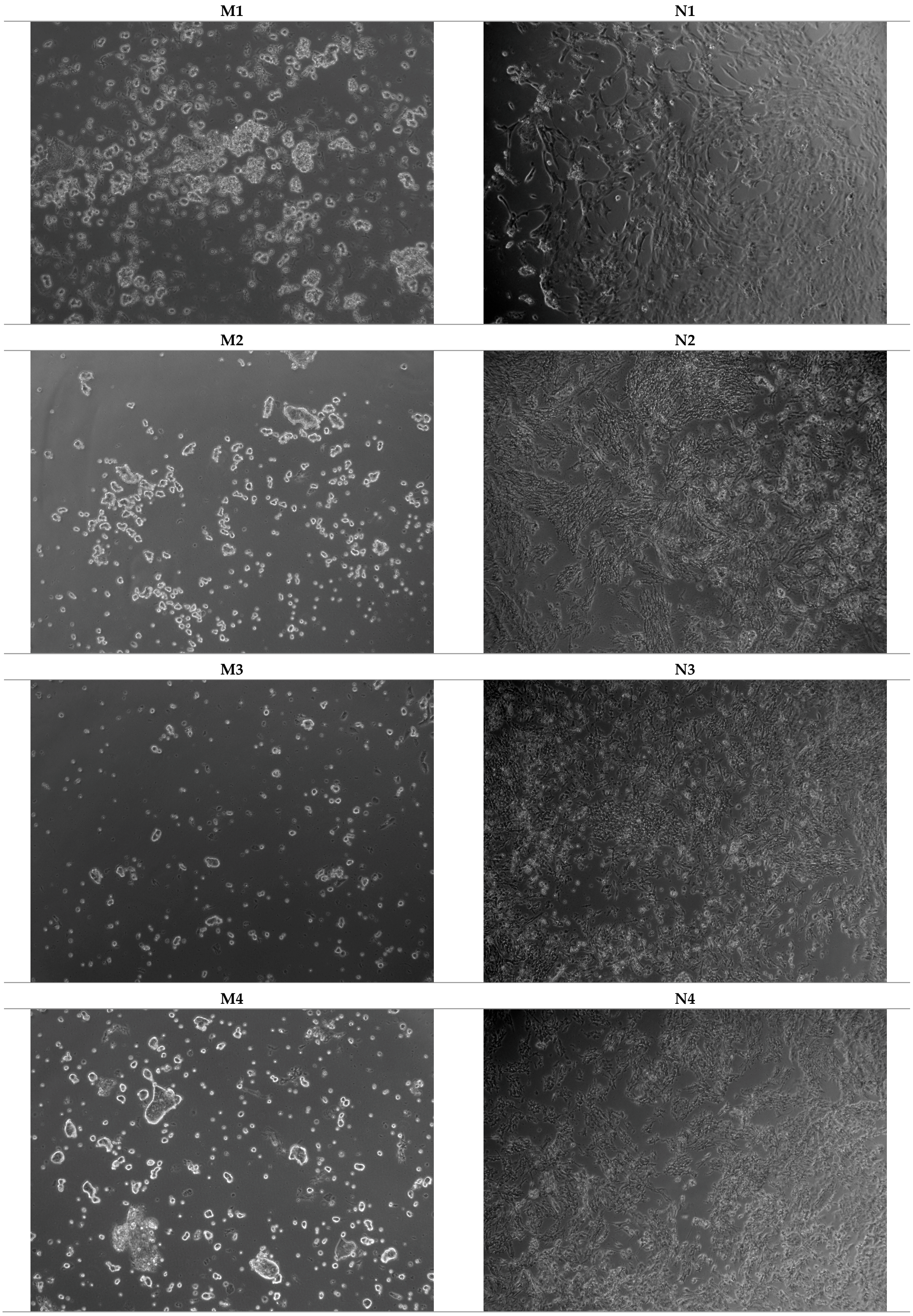

Figure S9. Phase contrast microscopy images taken at 96 hours with 10X objective
